# Metaproteomic Analysis of Fermented Vegetable Formulations with Lactic Acid Bacteria: A Comparative Study from Initial Stage to 15 Days of Production

**DOI:** 10.3390/foods14071148

**Published:** 2025-03-26

**Authors:** Narisa Rueangsri, Sittiruk Roytrakul, Chawanphat Muangnoi, Kullanart Tongkhao, Sudathip Sae-Tan, Khemmapas Treesuwan, Jintana Sirivarasai

**Affiliations:** 1 Doctoral Program in Nutrition, Faculty of Medicine Ramathibodi Hospital and Institute of Nutrition, Mahidol University, Bangkok 10400, Thailand; narisa.nr@gmail.com; 2 National Center for Genetic Engineering and Biotechnology (BIOTEC), National Science and Technology Development Agency, Pathum Thani 12120, Thailand; sittiruk@biotec.or.th; 3 Institute of Nutrition, Mahidol University, Nakhon Pathom 73170, Thailand; chawanphat.mua@mahidol.ac.th; 4 Department of Food Science and Technology, Faculty of Agro-Industry, Kasetsart University, Bangkok 10900, Thailand; kullanart.t@ku.ac.th (K.T.); fagists@ku.ac.th (S.S.-T.); 5 Institute of Food Research and Product Development, Kasetsart University, Bangkok 10900, Thailand; ifrkpt@ku.ac.th; 6 Nutrition Unit, Faculty of Medicine Ramathibodi Hospital, Mahidol University, Bangkok 10400, Thailand

**Keywords:** metaproteomics, metabolic pathway, lactic acid bacteria, fermented vegetables, functional food

## Abstract

Research in metagenomics and metaproteomics can reveal how microbiological interactions in fermented foods contribute to their health benefits. This study examined three types of fermented vegetables: a standard formulation, a probiotic formulation with Lacticaseibacillus rhamnosus GG, and a polyphenol formulation with vitexin from Mung bean seed coat. Measurements were taken at day 0 (after 36 h of fermentation at room temperature) and after 15 days. We applied 16S rRNA sequencing to evaluate microbial diversity and utilized LC-MS/MS to investigate the proteomic profiles of specific genera (*Lactobacillus* and *Weissella*) and species (*Lacticaseibacillus rhamnosus* and *Levilactobacillus brevis*) of lactic acid bacteria (LAB). All of these taxa demonstrated significant relative abundance between 0 and 15 days of fermentation in our metagenomic analysis. Our findings from principal component analysis and clustering analysis categorically distinguished protein expression patterns at various stages of fermentation. By comparing samples from day 0 to day 15, we identified proteins associated with DNA replication and repair mechanisms, including transcription elongation factor GreA, tRNA pseudouridine synthase B, and helicases. We also observed their roles in protein synthesis, which encompasses oxidoreductases and aspartokinase. Furthermore, we identified strong correlations of specific proteins across the three formulations with antioxidant markers. In conclusion, the results of this study decisively enhance our understanding of the role of the proteins related to specific LAB in fermented foods, highlighting their potential to improve texture, flavor, nutritional quality, and health benefits.

## 1. Introduction

Some fermented foods that possess beneficial nutritional properties and demonstrate health-related interactions with the human microbiome can be classified as functional foods. These foods play a significant role in managing metabolic syndrome due to their nutritional benefits, which include reducing inflammation, enhancing insulin sensitivity, and promoting gut health and lipid metabolism, potentially leading to reduced low-density-lipoprotein cholesterol and increased high-density-lipoprotein cholesterol levels [1]. The consumption of functional foods with proven cardiovascular benefits may play a role in reducing the risk of cardiovascular diseases (CVDs), as supported by scientific evidence [2]. Fermentation plays a crucial role in producing fermented vegetables. Fermentation enhances their nutritional profile by increasing the bioavailability of essential vitamins and conferring health-promoting properties, such as higher antioxidant potential. Additionally, certain fermentation processes can introduce beneficial probiotics, depending on factors such as microbial composition, fermentation conditions, and post-fermentation handling [3]. A previous study found that *Weissella*, a genus of lactic acid bacteria, possesses probiotic and antioxidant properties in kimchi, exhibiting the suppression of the expression of pro-inflammatory cytokines, interleukin (IL)-1β, IL-6, and tumor necrosis factor-α in LPS-induced RAW 264.7 cells [4]. Another study explored the beneficial effects of kimchi in protecting against liver damage caused by a high-cholesterol diet in mice with the knockout of a low-density-lipoprotein receptor. The results indicated that kimchi methanol extracts effectively decreased the expression of genes related to fatty acid synthesis while enhancing the expression of those involved in beta-oxidation. This suggests that kimchi may play a positive role in regulating lipid metabolism and supporting liver health [5]. Our current study indicates that there is microbial diversity in the taxonomic composition of various formulas of fermented vegetables, including standard formulas and those enhanced with probiotics and polyphenols [6]. The formulas prominently feature various bacteria, particularly lactic acid bacteria (LAB), including the main genera such as *Weissella*, followed by *Pediococcus, Leuconostoc*, and *Lactobacillus* [6]. Specifically, it was reported that the supplementation of fermented vegetables with *L. rhamnosus* GG and vitexin can increase the abundance of LAB, suppress pathogenic bacteria, and enhance antioxidant activity [6]. Further research examining the relationship between these probiotics and the potential activities of fermented foods, particularly the mechanisms linked to their health benefits, is required.

Proteomic studies on fermented vegetables are crucial for understanding the molecular mechanisms that underpin the health benefits associated with their probiotics and bioactive compounds. By analyzing the protein profiles, it is possible to identify important proteins with differential expression and metabolic pathways contributing to the effectiveness of probiotics, such as their role in gut health and immune modulation [7]. A comprehensive proteomic study of fermented foods, with descriptive analyses, has provided valuable insights into the metabolic activities of microbial communities under specific conditions. Comparative analyses have revealed the diverse taxonomic composition and functional capabilities of these microbial communities across various processes and formulations, highlighting their significance in food production [8]. For example, a total of 3493 proteins were identified in traditionally fermented *dajiang*, a fermented soybean paste, compared to 1987 proteins in commercially fermented *dajiang*. The microbial sources of these proteins were categorized, and analysis indicated that the majority of them are linked to five primary metabolic pathways: the pentose phosphate pathway, starch metabolism, sucrose metabolism, galactose metabolism, and pyruvate metabolism [9]. Moreover, a comparative proteomic analysis of kimchi samples manufactured in China and Korea showed significant differences in the numbers of proteins related to information storage and processing, cellular processes and signaling, and carbohydrate metabolism [10]. Another study effectively highlighted the proteomic characteristics and fermentation dynamics of indigenous bacteria isolated from traditional Mihaliç cheese, revealing significant proteolytic changes associated with fermentation that led to the production of free amino acids that enhance nutritional value, the formation of unique aromatic compounds, and the refinement of the cheese’s textural and sensory properties [11].

Despite the large number of studies described above, there has been limited research involving the metaproteomic analysis of fermented vegetables during various storage times. This highlights a gap in our understanding of how microbial communities and their protein expression change throughout fermentation and storage, particularly regarding the impact of storage duration on the metaproteome of LAB in different formulations. To help bridge this research gap, this study investigated how storage conditions and duration affect the protein expression and functional diversity of LAB. The obtained findings could enhance our knowledge of these processes and their implications for food science and health.

## 2. Materials and Methods

### 2.1. Process of Fermented Vegetable Production

This study outlines a fermentation procedure utilizing fresh Chinese cabbage (Makro, Bangkok, Thailand). The cabbage was meticulously washed to remove any undesirable components and subsequently trimmed to ensure quality. The proportion of cabbage in the recipe ranged from 55% to 90%. After washing, low-sodium salt (Good Life, Nakornpathom, Thailand) was added at a rate corresponding to 1% to 10% of the cabbage’s weight. This mixture was left to stand at room temperature for approximately 5 h. Next, a variety of seasonings were added, including red pepper powder (Gochugaru, Bangkok, Thailand) (0.1% to 7%), low-sodium fish sauce (Good Life, Nakornpathom, Thailand) (1% to 9%), onion (Makro, Bangkok, Thailand) (1% to 10%), ginger (Makro, Bangkok, Thailand) (0.1% to 5%), and garlic (Makro, Bangkok, Thailand) (1% to 5%). Further additions included water (Nestle, Phra Nakhon Si Ayutthaya, Thailand) (1% to 25%), low-calorie sugar (Sucrose, Mitr Phol, Suphanburi, Thailand) (55% to 90%), glutinous rice flour (1% to 5%), and chopped carrots (Erawan brand, Nakornpathom, Thailand) (0.1%). In this study, each ingredient was used at a selected concentration, which cannot be disclosed for reasons of intellectual property. The mixture was then appropriately seasoned and allowed to ferment in sealed containers at room temperature for 36 h.

A comprehensive overview of the methods used for preparing probiotic- and polyphenol-fermented vegetables is provided in our previous report [6]. The standard formula has already described above. To prepare probiotic-fermented vegetables, 0.01 g of lyophilized *Lactobacillus rhamnosus LGG*^®^ (*LGG*) purchased from Chr. Hansen A/S (Hoersholm, Denmark) was mixed into the fermented vegetables to achieve a final concentration of 10^8^ CFU per gram. For the preparation of vitexin-fermented vegetables, the polyphenol extract from the mung bean seed coat was dissolved in the liquid from the fermented vegetables, resulting in a final concentration of 5.5 mg of vitexin per 50 g of fermented vegetable [6]. In this study, we focused on three distinct formulations (standard fermented vegetables, N group; probiotic-fermented vegetables, P group; and vitexin-fermented vegetables, V group), each carefully maintained at 4 °C for 15 days. Following this period of storage, we evaluated the microbial communities and protein profiles in the fermented vegetables.

### 2.2. Measurement of the Antioxidant Activity of Fermented Vegetables

Samples were freeze-dried using a Kinetic Engineering Co., Ltd. LD-0.5 machine. Then, 1 g of the powdered fermented vegetable samples was extracted with 15 mL of distilled water, based on an adapted method from Park et al. [12]. The mixture was shaken at room temperature for 12 h and filtered through a Whatman filter paper. Finally, the total phenolic content (TPC), ferric-reducing antioxidant power (FRAP), oxygen radical absorbance capacity (ORAC), and DPPH radical scavenging activity (DPPH) were measured.

#### 2.2.1. Total Phenolic Content (TPC)

The total phenolic content (TPC) was quantified using the Folin–Ciocalteau method [13]. Specifically, a reaction mixture was prepared by combining 160 μL of distilled water, 10 μL of the sample extract, 20 μL of a saturated sodium carbonate solution (75% *w*/*v*), and 10 μL of Folin–Ciocalteau reagent within a 96-well plate. Following incubation at room temperature for a duration of 90 min, the absorbance was measured at a wavelength of 750 nm. The TPC was determined by referencing a standard curve for gallic acid, with the results expressed in micrograms of gallic acid equivalent (GAE) per gram of sample.

#### 2.2.2. Ferric-Reducing Antioxidant Power (FRAP)

The FRAP value of the samples was assessed using the method established by Halvorsen et al. [14]. Initially, a FRAP reagent solution was formulated by combining 10 mL of acetate buffer (300 mM) and diluting it to a final volume of 1000 mL with distilled water. Subsequently, a mixture was prepared by adding 1 mL of 2,4,6-Tris(2-pyridyl)-s-triazine (10 mM) dissolved in hydrochloric acid (40 mM) with 1 mL of ferric chloride hexahydrate (20 mM) in distilled water. Following this preparation, 20 μL of the sample extract and 150 μL of the FRAP reagent were introduced into a 96-well plate. The plate was incubated for 8 min at room temperature to ensure optimal reaction conditions. Finally, the FRAP value was measured at 600 nm, utilizing a standard curve for Trolox to ensure accuracy.

#### 2.2.3. Oxygen Radical Absorbance Capacity (ORAC)

ORAC was measured using the method described by Huang et al. [15]. The ORAC working buffer solution was prepared by mixing 603 mL of potassium dihydrogen phosphate (0.75 M) with 351 mL of dipotassium hydrogen phosphate (0.75 M) and then diluting 100 mL of this stock solution with 900 mL of distilled water. The fluorescein working solution was prepared by making a concentrate solution of 0.045 g fluorescein sodium salt in 100 mL of ORAC working buffer. This concentrate was diluted by mixing 50 μL with 9950 μL of the buffer. Additionally, 1.95 mL of fluorescein stock solution was diluted with 98.05 mL of the buffer. In a 96-well plate, 25 μL of the sample extract, 25 μL of standard Trolox solution, 25 μL of blank (ORAC working buffer), and 150 μL of fluorescein working solution were added. The plate was incubated at 37 °C for 30 min, after which 25 μL of 2ʹ-azobis(2-amidinopropane) dihydrochloride (AAPH) was added. Absorbance was measured every minute for 150 min using a fluorescein microplate reader at an excitation wavelength of 485 nm and an emission wavelength of 540 nm. ORAC values were calculated using a Trolox standard curve and expressed as micromoles of Trolox equivalent per gram of sample.

#### 2.2.4. DPPH Radical Scavenging Activity (DPPH)

The DPPH (2,2-diphenyl-1-picrylhydrazyl) free radical-scavenging activity was evaluated according to the method by Amarowicz et al. [16]. In this procedure, 20 μL of the sample extract was mixed with 100 μL of a DPPH reagent solution at a concentration of 150 μM in a 96-well microplate. After thorough mixing, the plate was incubated at room temperature for 30 min to facilitate the reaction. Following this incubation period, the absorbance of the solution was measured at a wavelength of 520 nm using a spectrophotometer. The antioxidant activity, indicated by a reduction in absorbance, was quantified against a standard curve established with Trolox, a water-soluble vitamin E analog, which serves as a benchmark for assessing the scavenging capacity of the sample.

### 2.3. DNA Extraction and Sequencing

DNA extraction from the microorganisms was conducted on the fermented vegetables using the DNeasy PowerSoil Pro kit developed by Qiagen (USA). To ensure the integrity and suitability of the extracted DNA, its quantity and quality were assessed using both Nanodrop spectrophotometry and electrophoresis. The next step involved the amplification of the V4 hypervariable region of the 16S rRNA gene via polymerase chain reaction (PCR). For this process, specific primers were used—515 F and 806 R—alongside a 2× KAPA hot-start ready mix, which enhances the precision of the amplification. The PCR protocol began with a 3 min denaturation step at 94 °C, effectively separating the DNA strands. This was followed by 25 amplification cycles, which comprised 20 s at 98 °C to ensure complete denaturation, 30 s at 55 °C for annealing of the primers, and 30 s at 72 °C for extension. The amplification concluded with a final extension phase at 72 °C lasting 5 min to ensure the complete synthesis of the PCR products.

Following amplification, the 16S samples underwent purification using AMPure XP beads, a crucial step to eliminate any residual contaminants that may interfere with downstream applications. They were then tagged with a Nextera XT index kit, facilitating the identification of individual samples during sequencing. This was followed by eight additional cycles of PCR under the same conditions to enhance the yield of the desired products. The purified and indexed PCR products were then carefully pooled together in preparation for cluster formation, as a key step in next-generation sequencing. This culminated in the sequencing of the samples using the Illumina^®^ MiSeq™ platform, employing a method that generates 250 bp paired-end reads, thereby enabling a comprehensive analysis of the microbial diversity in the fermented vegetables.

### 2.4. Microbiome Bioinformatic Analysis

Microbiome bioinformatics was performed with QIIME 2 2022.2 [17]. The raw sequence data were demultiplexed using the q2-demux plugin, and reads with expected errors (maxEE) exceeding 3.0 were discarded by employing denoising software, DA-DA2 (via q2-dada2; QIIME 2 2022.2); 16S sequences belonging to chloroplasts and mitochondria were also removed. Subsequently, the alpha diversity of the microbiome, which refers to the diversity of species within a sample in terms of species richness (number of different species) and evenness (distribution of individuals among those species), was analyzed using the Simpson index. Following this, beta-diversity metrics (Jaccard index) were computed using the q2-diversity tool, with samples filtered to a specified number of reads. Subsequently, principal coordinate analysis (PCoA) was performed to visualize the variations in community composition among the samples. In PCoA, a distance matrix derived from the Jaccard coefficients is constructed, allowing the representation of samples in a low-dimensional space where similar samples are located more closely together. Statistical analysis of beta diversity was performed using PERMANOVA (number of permutations = 999), and differential abundance analysis of the microbiota was conducted using nonparametric factorial Kruskal–Wallis rank-sum tests, followed by the Wilcoxon rank-sum test to correct for multiple comparisons. The taxonomic analysis involved the use of the SILVA database, a comprehensive resource for ribosomal RNA sequences, in conjunction with the naive Bayes classifier, a probabilistic machine learning algorithm. This combination was employed to accurately determine the taxonomic classification of sequences obtained from amplicon sequencing variants, thereby providing a deeper understanding of the microbial diversity in the sample.

### 2.5. Proteomic Analysis of All Formulas of Fermented Vegetables by LC-MS/MS

Liquid chromatography–tandem mass spectrometry (LC/MS-MS) for shotgun proteomics.

The protein concentrations of the fermented vegetable samples were quantified using the Lowry assay with bovine serum albumin (BSA) as a standard [18]. The protein samples (5 µg) underwent in-solution digestion. The samples were dissolved in 10 mM ammonium bicarbonate (AMBIC), followed by a reduction in disulfide bonds with 5 mM dithiothreitol (DTT) in 10 mM AMBIC at 60 °C for 1 h. Subsequently, sulfhydryl groups were alkylated with 15 mM iodoacetamide (IAA) in 10 mM AMBIC at room temperature for 45 min in the dark. Proteins were digested with sequencing-grade porcine trypsin at a 1:20 enzyme-to-substrate ratio for 16 h at 37 °C. The resulting tryptic peptides were dried using a vacuum concentrator and reconstituted in 0.1% formic acid for analysis via nano-liquid chromatography–tandem mass spectrometry (nanoLC-MS/MS).

The tryptic peptide samples were prepared for analysis using an Ultimate 3000 Nano/Capillary LC System (Thermo Scientific, Cambridge, UK) coupled to a hybrid quadrupole Q-ToF impact II™ mass spectrometer (Bruker Daltonics, Billerica, MA, USA) equipped with a Nano-CaptiveSpray ion source. Briefly, 1 µL of peptide digest was loaded onto a µ-Precolumn (300 µm i.d. × 5 mm, C18 PepMap 100, 5 µm, 100 Å; Thermo Scientific, UK) for enrichment and subsequently separated on a 75 µm i.d. × 15 cm analytical column packed with Acclaim PepMap RSLC C18 material (2 µm, 100 Å, nanoViper; Thermo Scientific, UK). The analytical column was maintained at 60 °C within a thermostat oven. The mobile phase consisted of solvent A (0.1% formic acid in water) and solvent B (0.1% formic acid in 80% acetonitrile). Peptides were eluted using a linear gradient of 5–55% of solvent B over 30 min at a constant flow rate of 0.30 µL/min. Electrospray ionization was conducted at 1.6 kV using the CaptiveSpray ion source, with nitrogen as the drying gas at a flow rate of approximately 50 L/h. Collision-induced dissociation (CID) was performed with nitrogen as the collision gas, and product ion mass spectra were acquired in positive-ion mode. Full mass spectra (MS) and tandem mass spectra (MS/MS) were recorded at a scan rate of 2 Hz over an *m*/*z* range of 150–2200. Collision energy was adjusted to 10 eV optimized according to the *m*/*z* of the precursor ions. Each sample was analyzed in triplicate by LC-MS.

Bioinformatics and data analysis

Protein quantification for individual samples was conducted using MaxQuant version 2.2.0.0 with the Andromeda search engine employed to match MS/MS spectra to the UniProt bacterial database [19]. Label-free quantification was performed using the default MaxQuant settings, including a maximum of two missed cleavages, a mass tolerance of 0.6 Da for the main search, and trypsin as the designated proteolytic enzyme. Carbamidomethylation of cysteine was set as a fixed modification, while methionine oxidation and N-terminal acetylation were included as variable modifications. Peptides were required to have a minimum length of seven amino acids and at least one unique peptide to qualify for protein identification. Proteins were considered identified if they were associated with at least two peptides, including one unique peptide. A 1% false discovery rate (FDR) for protein identification was enforced and calculated using a reverse sequence search. The maximum number of modifications allowed per peptide was set to five.

Protein sequences from the bacterial proteome were downloaded from UniProt (as of 18 January 2024) and used as the FASTA search database. The raw MS/MS data and accompanying analyses are publicly available in the ProteomeXchange Consortium via the jPOST partner repository under the identifier JPST003461 with the dataset identifier PXD057742.

Data analysis

Measurements were taken at day 0 (after 36 h of fermentation at room temperature) and after 15 days. For the metaproteomic analysis of individual LAB genera and species, we selected two genera: *Lactobacillus* and *Weissella*, and two species: *Lacticaseibacillus rhamnosus* and *Levilactobacillus brevis*. All of these demonstrated significant relative abundance in our previous analysis [6]. Visualization and statistical analyses of the LC-MS data were performed using MetaboAnalyst version 6.0, incorporating partial least squares discriminant analysis (PLS-DA), the Variable Importance in Projection (VIP) score (a plot of the most important discriminant proteins by PLS-DA) and one-way analysis of variance with a significance threshold set at *p* < 0.05 [20]. A Venn diagram was used to highlight the differences between protein lists obtained from distinct differential analyses [21]. Overall data from Venn diagrams were described in Supplementary Figures S1–S4. Functional annotation and biological organization of differentially expressed proteins were carried out using ShinyGO version 0.77 [22]. Functional interaction networks, including common and predicted associations between identified proteins and small molecules, were analyzed using the STITCH database version 5 [23].

## 3. Results

### 3.1. Microbial Diversity of Fermented Foods Classified by Formula

**Figure 1:**
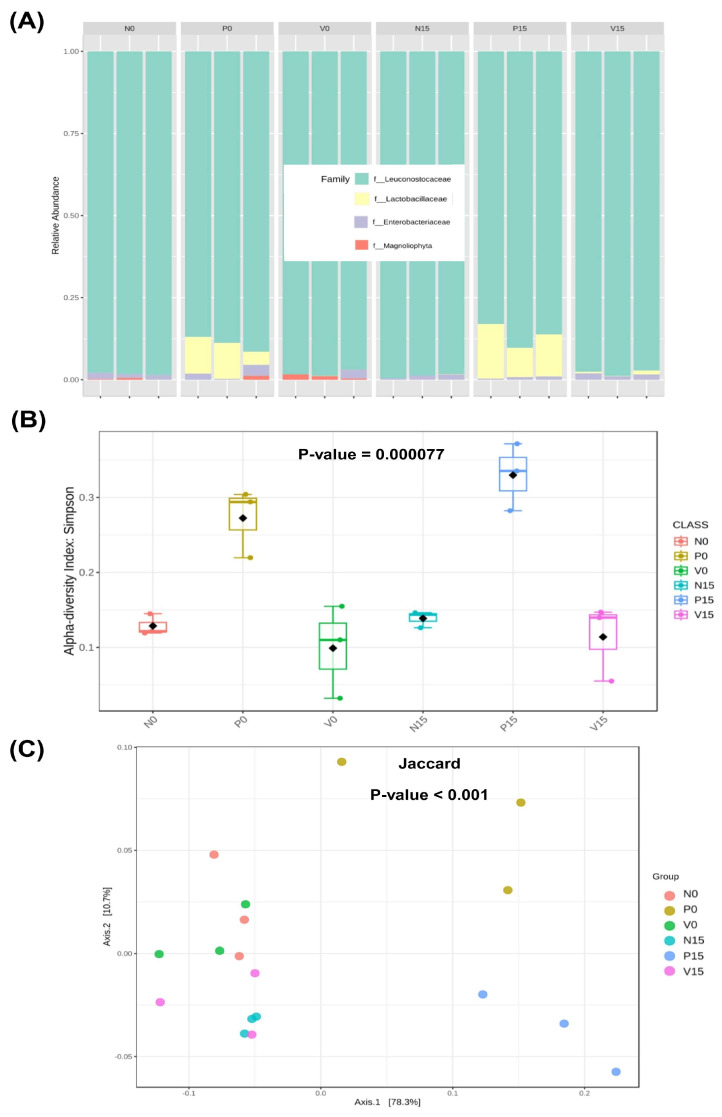
Relative abundance of microbiome among different fermented vegetable formulas (standard fermented vegetables, N group; probiotic-fermented vegetables, P group; and vitexin-fermented vegetables, V group) (**A**); (**B**) alpha diversity as Simpson index (B); and beta diversity plots based on Jaccard indice (**C**).

In the analyses of all three formulas of fermented vegetables at day 0 and day 15, we found high relative abundances of *Leuconostocaceae* and *Lactobacillaceae*, with substantially low relative abundances of *Enterobacteriaceae* and *Erwiniaceae*, as shown in Figure 1A. The Simpson index reflecting alpha diversity and species richness was significantly different (*p* = 0.000077) across the fermented vegetable groups at other times, indicating differences in the community structure (number and proportions) of bacteria (Figure 1B). PCoA based on Jaccard distance was used to compare the β diversity. The results showed that there were significant differences (*p* < 0.001) in the microbial diversity of fermented foods with different formulas (Figure 1C). According to our previous report [6], significant differences in the abundances of some bacterial taxa between day 0 and day 15 were found, including *Lacticaseibacillus rhamnosus* in the probiotic-fermented vegetable formula and *Lactobacillus*, *Weissella*, and *Lactobacillus brevis* in the polyphenol-fermented vegetable formula (data are presented in Supplementary Table S1) [6]. The statistical analysis revealed the following key findings: Standard Formula (N group) showed no significant microbial changes after FDR correction. Although *Pediococcus* exhibited a lower *p*-value (*p* = 0.023), it did not reach statistical significance (FDR = 0.243). Probiotic Formula (P group) led to significant increases in *Lacticaseibacillus rhamnosus* (FDR = 0.0115) and *Pediococcus* (FDR = 0.0153), suggesting a potential probiotic effect of this formulation. Vitexin Formula (V group) exhibited the most substantial microbial shifts, with significant increases in *Levilactobacillus brevis* (FDR = 0.000155), Lactobacillus (FDR = 0.00645), and *Weissella* (FDR = 0.0156), indicating a strong influence on specific bacteria. These results indicated a significant diversity of microbial composition among the different formulas, especially in terms of LAB. Therefore, we further analyzed the protein profiles associated with both genera and species of LAB at the two time points.

### 3.2. Metaproteomic Analysis of Individual LAB Genera and Species

#### 3.2.1. *Genus Lactobacillus*

**Figure 2:**
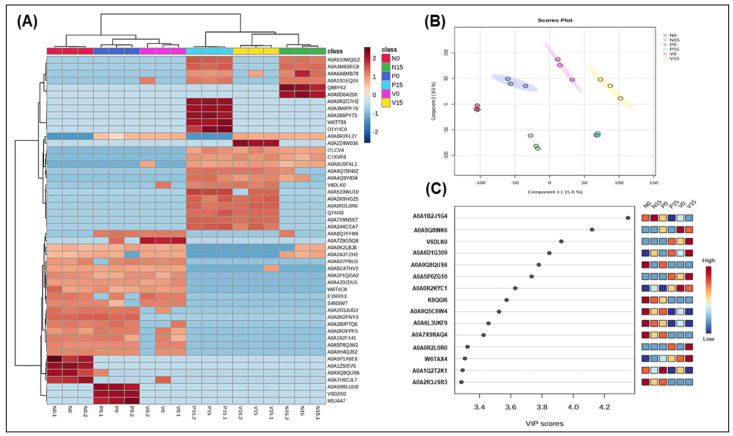
Hierarchical clustering analysis of protein expression related to genus *Lactobacillus* among 6 groups of fermented vegetables (N0 and N15; Standard formula at day 0 and day 15, P0 and P15; Probiotic formula at day 0 and day 15, V0 and V15; Vitexin formula at day 0 and day 15). The color scale illustrates the relative concentration of the differential proteins. Dark red represents a relatively high concentration, while dark blue represents a relatively low concentration (**A**); Score plots of PLS-DA (**B**) and variable importance in projection (VIP) with high score of five proteins (**C**). Analysis was performed by MetaboAnalyst 5.0 program.

Figure 2 described the proteomic analysis of fermented vegetables related to genus Lactobacillus between three groups of fermented vegetables at two time points, including hierarchical clustering analysis showing protein expression patterns across six fermented vegetable groups, highlighting similarities and differences in the fermentation profiles; the PLS-DA score plot illustrating group separation based on proteomic profiles, indicating distinct protein expression patterns; and the VIP plot identifying the top five proteins contributing to group differentiation.

The hierarchical clustering heatmap (Figure 2A) effectively highlights the relative abundance of proteins associated with the genus *Lactobacillus* in six groups of fermented vegetables at days 0 and 15. At day 0, the clustering pattern reflects a significant degree of similarity among all groups, as evidenced by a uniform distribution of colors. Notably, some groups show a promising increase in *Lactobacillus*-related proteins, indicated by clusters enriched in red, which denotes higher protein abundance. In contrast, other groups reveal a decrease in protein expression, as illustrated by the blue-shaded regions. The following proteins were identified exclusively in the standard formula on day 0 and were not detected on day 15: A0A5P1X6E6 (Protein RecA), A0A1Z5IEV5 (UDP-N-acetylglucosamine 1-carboxyvinyltransferase), A0A9Q8QUS6 (Polyphosphate kinase), and A0A7H9CJL7 (Glycosyltransferase family 4 protein). On the other hand, A0A5R8LUU0 (ATP-binding cassette domain-containing protein), V5DZK0 (tRNA(Ile)-lysidine synthase), and M5J4A7 (D-alanine ligase) were found only in the probiotic formula on day 0. Additionally, proteins such as A0A4Z0JDU1 (IS110 family transposase) and W6T4C8 (2,5-diketo-D-gluconic acid reductase) were present in all three formulas on day 0, but were absent by day 15. Notably, a significant number of proteins were identified only on day 15, including Q88YK2 (phosphatidylglycerol--prolipoprotein diacylglyceryl transferase) and A0A0D6A210 (DNA polymerase IV) for the standard formula; A0A2Z4W036 (Ribonuclease Y) for the vitexin formula; and A0A0R2G7H2 (Diadenosine tetraphosphatase-like protein), A0A3M0PF76 (Primosomal protein N), A0A386PY73 (LysR family transcriptional regulator), and W6T7599 (Major facilitator transporter). These changes in protein expression may reflect the effects of different time points, probiotic supplementation, and the presence of bioactive compounds, such as vitexin.

The multivariate analysis technique of partial least squares discriminant analysis (PLS-DA) was used to classify samples and identify the most important variables for distinguishing between the fermented vegetable samples (N0, N15, P0, P15, V0, and V15), as illustrated in Figure 2B. The PLS-DA model pinpointed the most discriminative protein variables within the dataset of the three formulas. The PLS-DA score plot clearly revealed that fermentation significantly alters protein expression between day 0 and day 15, indicating time-dependent changes in protein levels. Additionally, the most critical protein, determined by VIP scores, plays a key role in this separation. The visualization effectively demonstrates the time-dependent proteomic changes in fermented vegetables.

The VIP scores were calculated for each variable, which provided critical insights into the discriminative features of the dataset, with the threshold set to VIP > 1. The top 10 proteins identified by VIP scores are summarized in Figure 2C. Notably, A0A1B2J1G4 exhibited the highest VIP score, indicating its strong influence on the model. Conversely, protein had a VIP score of 0.5, suggesting it is minimally important for differentiating between the groups. In this study, the proteins with high VIP scores (ranging from 3.2 to 4.6) corresponded to A0A1B2J1G4 (transcription elongation factor GreA), A0A9Q8INK6 (uncharacterized protein), V6DLK0 (uncharacterized protein), A0A6D1G309 (deleted protein), and A0A9Q8QUS6 (polyphosphate kinase-phosphorylation).

#### 3.2.2. Genus Weissella

**Figure 3:**
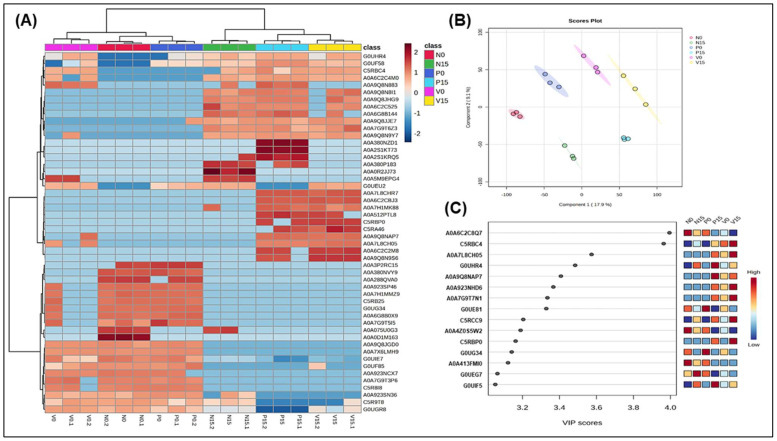
Hierarchical clustering analysis of protein expression related to genus *Weissella* among 6 groups of fermented vegetables (**A**); Score plots of PLS-DA (**B**) and variable importance in projection (VIP) with high score of five proteins (**C**). Analysis was performed by MetaboAnalyst 5.0 program. (N0 and N15; Standard formula at day 0 and day 15, P0 and P15; Probiotic formula at day 0 and day 15, V0 and V15; Vitexin formula at day 0 and day 15).

In Figure 3, we conducted a hierarchical clustering analysis to examine the differential protein profiles of the genus *Weissella*. This analysis revealed significant differences in protein expression among the various groups. Notably, some proteins were significantly upregulated in all fermented vegetable groups on day 0 but showed a slight downregulation by day 15 (Figure 3A). The proteins that exhibited this trend included A0A9Q8JGD0 (NgoFVII family restriction endonuclease) and A0A7X6LMH9 (aminoacyltransferase). In contrast, A0A7L8CHR7 and A0A6C2CBJ3 (both ABC transporter permeases) were significantly upregulated in three of the formulas on day 15. When examining each formula specifically, we found that in the standard formula, A0A0D1M163 (VWA-like domain-containing protein) was upregulated on day 0 but downregulated on day 15. For the probiotic formula, A0A380NVY9 (ribonuclease R) showed the same pattern, while in the vitexin formula, A0A9Q8N883 (ABC transporter substrate-binding protein) was also upregulated on day 0 but downregulated by day 15. From Figure 3B (PLS-DA score plot), the main findings included the six fermented vegetable groups (N0, N15, P0, P15, V0, V15) are clearly separated along Component 1 (15.6%) and Component 2 (11.4%), indicating distinct proteomic profiles between groups. The clustering suggests that different conditions (e.g., time and formulas) significantly influence protein expression. VIP scores in PLS-DA help identify which proteins contribute most to the separation of groups, providing insights into key metabolic and microbial transformations occurring during fermentation. The high VIP scores of A0A6C2C8Q7 (oxidoreductase) and C5RBC4 (tRNA pseudouridine synthase B) suggested their critical roles in fermentation-associated proteomic shifts, making them potential biomarkers for studying the impact of *Weissella* in the fermentation process (Figure 3C).

#### 3.2.3. Species *Lacticaseibacillus rhamnosus*

**Figure 4:**
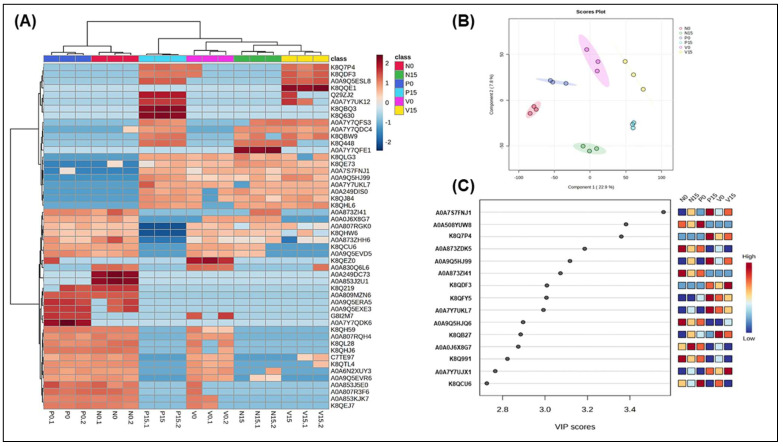
Hierarchical clustering analysis of protein expression related to *Lacticaseibacillus rhamnosus* among 6 groups of fermented vegetables (**A**); Score plots of PLS-DA (**B**) and variable importance in projection (VIP) with high score of five proteins (**C**). Analysis was performed by MetaboAnalyst 5.0 program. (N0 and N15; Standard formula at day 0 and day 15, P0 and P15; Probiotic formula at day 0 and day 15, V0 and V15; Vitexin formula at day 0 and day 15).

In Figure 4, we present a proteomic analysis of *Lacticaseibacillus rhamnosus* across six groups of fermented vegetables, utilizing hierarchical clustering (4A), partial least squares discriminant analysis (PLS-DA) (4B), and variable importance in projection (VIP) analysis (4C). The heatmap displays the differential expression of proteins across the six fermentation groups: N0, N15, P0, P15, V0, and V15. Two proteins, K8QH59 (cell surface hydrolase, membrane-bound) and A0A807RQH4 (Tyrosine–tRNA ligase), were significantly upregulated at day 0 but downregulated by day 15 across all three formulas. In the standard formula, A0A249DC73 (ADP-ribose pyrophosphatase) and A0A853J2U1 (Uncharacterized protein) exhibited low significant expression at day 15 compared to day 0. Additionally, A0A809MZN6 (uncharacterized protein) showed similar results in the probiotic formula. In the vitexin formula, several proteins demonstrated low expression at both day 0 and day 15, including A0A809MZN6 (uncharacterized protein), K8Q219 (poly(glycerol-phosphate) alpha-glucosyltransferase), A0A853J2U1 (uncharacterized protein), and A0A249DC73 (ADP-ribose pyrophosphatase). The score plot in Figure 4B visually represents the differences in proteomic profiles among the six fermented vegetable groups (N0, N15, P0, P15, V0, V15). The distinct clustering of groups along the two axes indicates that different times (day 0 vs. day 15) and treatment types (N, P, V) significantly affect proteomic variation. Additionally, the VIP scores highlight the proteins that play a crucial role in differentiating the groups based on their proteomic profiles. Three proteins of particular interest, with high VIP scores ranging from 2.6 to 3.6, include A0A7S7FNJ1 (phage minor tail protein), A0A5O8YUW8 (relaxase), and K8Q7P4 (L-proline glycine betaine-binding ABC transporter protein; ProX) (Figure 4C).

**Figure 5:**
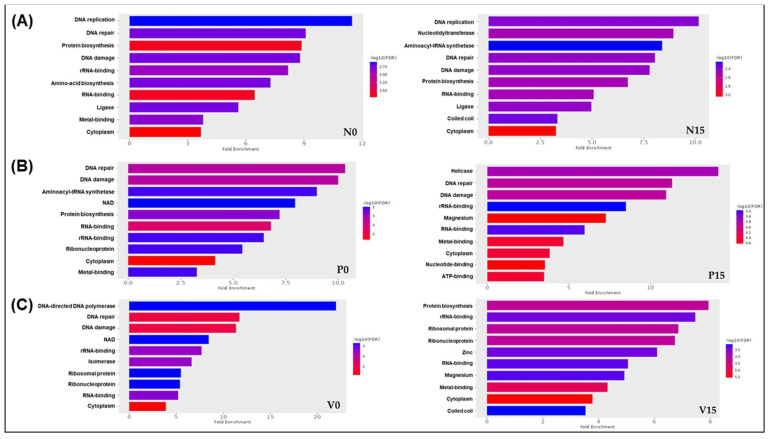
ShinyGo enrichment analysis of fermented vegetables related to *Lacticaseibacillus rhamnosus* among 6 groups of fermented vegetables; Standard formula day 0 and day 15 (**A**), Probiotic formula day 0 and day 15 (**B**); Vitexin formula day 0 and day 15 (**C**). (N0 and N15; Standard formula at day 0 and day 15, P0 and P15; Probiotic formula at day 0 and day 15, V0 and V15; Vitexin formula at day 0 and day 15).

We carried out a comprehensive analysis of fold enrichment to gain insights into the potential associations between the identified proteins across the six groups of fermented vegetables and specific biological processes. By using ShinyGO 0.88 software and applying a *p*-value cut-off (FDR) of <0.05, we obtained valuable insights, as illustrated in Figure 5A–C. Our findings highlighted distinct differences in UniProt-GO classifications among the groups. For the standard formula, we identified significant enrichments at day 0 for biological processes including DNA replication, DNA repair, and protein biosynthesis. By day 15, the notable processes had shifted to nucleotidyltransferases and aminoacyl-tRNA synthetase (Figure 5A). In the case of the probiotic formula, we observed that, at day 0, key enriched gene ontology categories included DNA repair, DNA damage, and aminoacyl-tRNA synthetase. By day 15, these categories had shifted to helicase, DNA repair, and DNA damage (Figure 5B). These changes underscore the dynamic nature of gene expression throughout the fermentation process. Furthermore, the enrichment analysis of the vitexin formula also provided insightful results. At day 0, we found significant processes such as DNA-directed DNA polymerase, DNA repair, and DNA damage. However, by day 15, these categories had shifted to highlight protein biosynthesis, rRNA binding, and ribosomal proteins (Figure 5C). These findings improve our understanding of biological processes in fermented vegetables and indicate directions for further research into the specific mechanisms behind these enrichments.

#### 3.2.4. Species *Levilactobacillus brevis*

**Figure 6:**
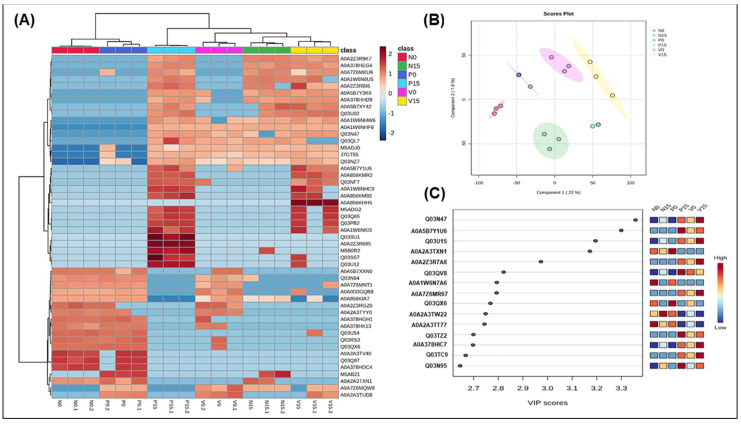
Hierarchical clustering analysis of protein expression related to *Levilactobacillus brevis* among 6 groups of fermented vegetables (**A**); Score plots of PLS-DA (**B**) and variable importance in projection (VIP) with high score of five proteins (**C**). Analysis was performed by MetaboAnalyst 5.0 program. (N0 and N15; Standard formula at day 0 and day 15, P0 and P15; Probiotic formula at day 0 and day 15, V0 and V15; Vitexin formula at day 0 and day 15).

The analysis of the differential protein profile associated with *Levilactobacillus brevis* revealed, through hierarchical clustering analysis, significant variations in protein expression across the six groups (Figure 6A). Notably, some proteins, such as A0A2Z3R9K7 (Fluoride-specific ion channel FluC) and A0A378HEG4 (DUF4145 domain-containing protein), were significantly upregulated in the all-fermented vegetable groups at day 15. Furthermore, we identified several significant upregulated proteins in the standard formula, including M5ADJ0 (Protein DltD), J7GT55 (Penicillin-binding protein), and Q03NZ7 (Asparagine–tRNA ligase). In the probiotic formula, the notable proteins included A0A2Z3R9K7 (fluoride-specific ion channel FluC) and A0A378HEG4 (DUF4145 domain-containing protein), as well as A0A2Z3R695 (Zn-dependent alcohol dehydrogenase) and Q03SS7 (DNA-directed DNA polymerase) in the vitexin formula. Figure 6B visualizes the distribution of sample groups based on their principal components, highlighting proteomic variations influenced by fermentation conditions. Samples from day 0 from all groups are well separated from those at day 15, suggesting that different timepoints significantly alters the proteomic profile of *Levilactobacillus brevis*. The VIP scores identify proteins that significantly contribute to the separation of groups in the PLS-DA model. Higher VIP scores indicate which proteins are most influential in distinguishing between fermentation groups. Several key proteins were found to have high VIP scores, ranging from 2.6 to 3.4. These include Q03N47 (nickase), A0A5B7Y1U6 (DNA helicase RecQ), and Q03U15 (alpha-glucosidase, family 31 of glycosyl hydrolases) (see Figure 6C).

**Figure 7:**
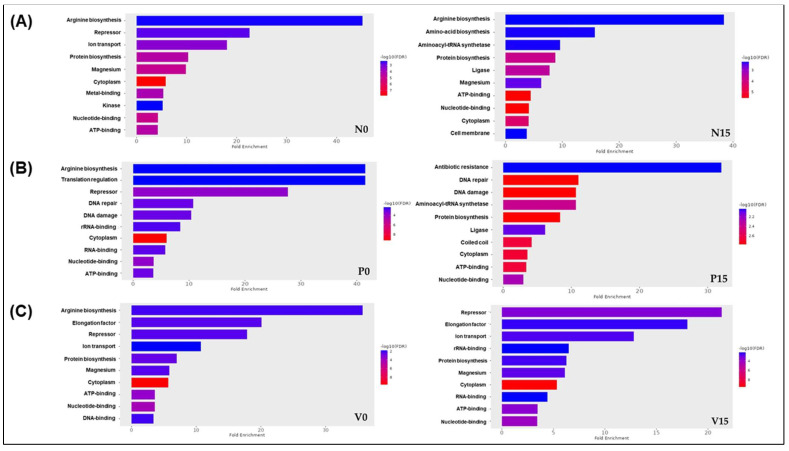
ShinyGo enrichment analysis of fermented vegetables related to *Levilactobacillus brevis* among 6 groups of fermented vegetables; Standard formula day 0 and day 15 (**A**), Probiotic formula day 0 and day 15 (**B**); Vitexin formula day 0 and day 15 (**C**). (N0 and N15; Standard formula at day 0 and day 15, P0 and P15; Probiotic formula at day 0 and day 15, V0 and V15; Vitexin formula at day 0 and day 15).

Our analysis of gene ontology (GO) enrichment concerning *Levilactobacillus brevis* revealed similar pathways across the three formulas at day 0, specifically pertaining to arginine biosynthesis and repressor activity. In contrast, the results observed at day 15 for the fermented vegetable samples indicated notable differences. The standard formula was associated with pathways related to arginine biosynthesis, amino acid biosynthesis, and aminoacyl-tRNA synthetase. The probiotic formula demonstrated pathways linked to antibiotic resistance, DNA repair, and DNA damage. Additionally, the vitexin formula was associated with pathways involving repressors, elongation factors, and iron transport (Figure 7A–C). These findings provide valuable insights into the functional roles of *Levilactobacillus brevis* across different formulations and timepoints.

#### 3.2.5. Assessment of Differences in Proteins Profiles Between Six Groups of Fermented Vegetables at Two Time Points (Day 0 and Day 15)

We applied ANOVA with a post hoc test (Tukey’s HSD) using MetaboAnalyst to statistically analyze and visualize variations in protein levels across the different fermented food groups over time. This method is valuable for deepening our understanding of the data. The analysis revealed significant differences in protein levels among the six groups of fermented vegetables, with 33, 30, 23, and 20 proteins associated with *Lactobacillus*, *Weissella*, *Lacticaseibacillus rhamnosus*, and *Levilactobacillus brevis*, respectively. These data are presented in detail in Supplementary Tables S2–S5. Table 1 presents the examples of five proteins that showed significant differences among the three formulas related to each genus.

#### 3.2.6. The Correlation Between Significant Proteins Identified Through ANOVA and the genera *Lactobacillus*, *Weissella*, *Lacticaseibacillus rhamnosus*, and *Levilactobacillus brevis* with Key Antioxidant Parameters

The heatmap visualization of significant proteins (*p* < 0.001) identified through ANOVA, highlighting their correlations with antioxidant activity markers, including TPC, DPPH, ORAC, and FRAP were described and are presented in Supplementary Figure S5. The analysis focuses on four bacterial groups: Lactobacillus, Weissella, Lacticaseibacillus rhamnosus, and Levilactobacillus brevis. For example, in our findings regarding Lactobacillus, A0A510WQG2 (ATP-dependent Clp protease ATP-binding subunit ClpC) demonstrated strong positive correlations with TPC, DPPH, and ORAC, while exhibiting a negative correlation with FRAP. The findings reveal distinct genus- and species-specific associations of these proteins with antioxidant activities, suggesting their potential role involving oxidative stress in fermented vegetables.

## 4. Discussion

In this study, we conducted a comprehensive analysis of metagenomic and metaproteomic data derived from fermented vegetables utilizing three distinct formulations. The results indicated significant relative abundances of the genera *Lactobacillus* and *Weissella* as well as the species *Lacticaseibacillus rhamnosus* and *Levilactobacillus brevis*. These findings not only align with those of previous research but also contribute valuable insights into the microbial communities present in fermented vegetable products [6]. The alpha diversity was found between the three categories of fermented vegetables which *Weissella* were dominant in all fermented vegetable formulas. *Weissella*, along with *Leuconostoc mesenteroides* and other lactic acid bacteria (LAB), are crucial in initiating the fermentation of vegetables, enhancing their nutritional and functional value while contributing to the development of beneficial probiotics during the fermentation process [24]. The detection of *Lactobacillus*, particularly *L*. *rhamnosus GG*, in probiotic-fermented vegetables highlights its dynamic role during fermentation. The increase in *L*. *rhamnosus* abundance at day 15 correlates with a decrease in *Weissella* spp., suggesting a complex interaction where Weissella may initially support *L*. *rhamnosus* growth through the production of extracellular polysaccharides (EPSs) that act as prebiotics [25]. In this study, L. brevis was identified as the predominant microorganism in vegetables fermented with vitexin. This dominance is likely due to the favorable pH conditions observed during fermentation, which ranged from 4.00 to 4.50 [6]. Such pH values are well within the optimal range for the growth of *L*. *brevis*, typically between 4.0 and 6.0, thus facilitating its proliferation in this specific fermentation environment [26]. The diverse range of probiotic strains in fermented foods is valuable and is influenced by food sources, fermentation processes, and environmental factors. Proteomics plays a critical role in elucidating the protein profiles of beneficial bacteria, deepening our understanding of their contributions to the fermentation process and overall health.

We present a detailed summary of our analysis related to the genus *Lactobacillus*, focusing on the most significant findings concerning the identified proteins and their corresponding VIP scores. We highlight those proteins with the highest VIP scores, as they are particularly relevant to our research objectives. Transcription elongation factor GreA and polyphosphate kinase (PPK) were identified as being related to the genus *Lactobacillus* (Figure 2C). GreA is a critical protein involved in transcription in bacteria. It enhances the transcription efficiency and plays a significant role in the regulation of gene expression [27]. Therefore, its activity is important for maintaining the metabolic functions and growth of *Lactobacillus* during the fermentation process. With regard to PPK, it is essential for polyphosphate synthesis. The disruption of this protein can reduce survival and stress resistance in probiotics [28]. Upon the comparison of the six groups of fermented vegetables, we noted two particularly important proteins: ATP-dependent Clp protease and repressor of the fructose operon. The level of the first of these was significantly higher in both the standard and probiotic formulas at day 15, while it was also positively correlated with antioxidant markers (TPC, DPPH, and ORAC). It plays a crucial role in protein quality control by degrading misfolded or damaged proteins and regulating the levels of certain cellular proteins. This protease is composed of a proteolytic core (ClpP) and ATPase components (ClpA or ClpX), which potentially provide antioxidant activity through their role in degrading oxidatively damaged proteins and maintaining cellular homeostasis [29]. Meanwhile, the repressor of the fructose operon in *Lactobacillus* may influence the antioxidant properties of fermented foods by modulating the expression of genes involved in carbohydrate metabolism [30] and the production of bioactive compounds. When the fructose operon is repressed, this can potentially lead to altered fermentation pathways, affecting the synthesis of metabolites such as lactic acid, which play a crucial role in the overall antioxidant capacity of the food product.

According to the findings for the genus *Weissella*, oxidoreductase and tRNA pseudouridine synthase B showed high VIP scores (Figure 3C). *Weissella* is a genus of lactic acid bacteria known for its role in fermentation processes, particularly in the production of various fermented foods. Oxidoreductases are enzymes that catalyze oxidation–reduction reactions, facilitating the transfer of electrons between molecules [26]. In the context of *Weissella*, oxidoreductases may be involved in metabolic pathways that contribute to the flavor, aroma, and preservation qualities of fermented products, as well as playing a role in bacterial survival and adaptation to different environments [31]. In addition, tRNA pseudouridine synthase B (TruB) exhibited a significant VIP score in the vitexin group on day 15. This enzyme is recognized for its essential function in RNA modification through pseudouridylation, which impacts tRNA stability and functionality [32]. Increased levels of TruB are likely to enhance tRNA stability, thereby improving the ability of *Weissella* to endure acidic, osmotic, and oxidative stress during the fermentation process. Moreover, the modifications to tRNA could influence protein synthesis, which may subsequently affect *Weissella*’s contributions to flavor and texture and its probiotic properties.

The ANOVA results also revealed remarkable increases in the levels of specific proteins across all three formulas by day 15, compared with the levels at day 0, including cardiolipin synthase (CL synthase), glycosyltransferase, SF3 helicase domain-containing protein, and aminopeptidase (Table S2). Cardiolipin has been noted for the roles it plays in bacterial membranes, particularly in membrane integrity, stability, and function [33]. The increased expression of cardiolipin synthase may reflect an adaptive response of *Weissella* to the fermentation process, potentially enhancing its resilience under the stress conditions that prevail during storage. Furthermore, the elevated cardiolipin level could have implications for the overall quality and probiotic properties of fermented food. Specifically, cardiolipin is known to influence cellular processes such as biofilm formation [34], which may enhance the textural and flavor characteristics of these food products. In addition, glycosyltransferases, which are critical enzymes in LAB that facilitate the synthesis of exopolysaccharides (EPSs), catalyze the transfer of sugar moieties from activated nucleotide sugars to elongating polysaccharide chains [30]. The actions of glycosyltransferases are pivotal in determining the structure, composition, and molecular weight of EPSs. These parameters are important in the context of the present study as they directly influence the texture, viscosity, and stability of fermented products, thereby enhancing their overall quality and consumer appeal [35].

Helicases are critical enzymes involved in nucleic acid metabolism, encompassing essential processes such as DNA replication, repair, recombination, and transcriptional regulation [36]. These enzymes are recognized for their role in facilitating bacterial stress responses by promoting DNA repair mechanisms and regulating the expression of stress-related genes [37]. The SF3 helicase family in particular has been implicated in RNA metabolism and the stress responses observed in bacteria [38]. The high expression of this protein in *Weissella* suggests its potential role in maintaining genetic stability and regulating gene expression in response to the environmental challenges associated with inhabiting fermented food. An additional highly expressed protein was aminopeptidase, with its high level suggesting that it plays a crucial role in proteolysis during the fermentation process. Aminopeptidases are essential enzymes responsible for hydrolyzing peptides into free amino acids, which make significant contributions to the development of the flavor, texture, and bioactive properties of fermented foods [39]. The elevated aminopeptidase levels detected in this study indicate the enhancement of the protein degradation process, which would potentially increase the availability of free amino acids which, in turn, influence the sensory and nutritional quality of the final product. In accordance with this, the association of *Weissella* with high aminopeptidase activity highlights its potential role in fermentation. *Weissella* species, commonly found in various fermented foods, are known for their ability to produce lactic acid and modify food texture and taste through enzymatic activity [40]. However, this enzyme showed a negative correlation with antioxidant parameters (Supplemetary Figure S5B). This may be explained by the excessive proteolysis leading to the degradation of bioactive peptides, reducing the overall antioxidant potential. Additionally, the presence of pro-oxidant amino acids such as tyrosine and methionine may lead to increased lipid oxidation in fermented foods, counteracting any residual antioxidant effects [41]. Finally, DUF536 domain-containing protein, which was expressed at high levels in all fermented vegetable groups at day 15 compared with the levels at day 0, showed positive correlations with antioxidant parameters, although no research on the association of this protein with fermented food has previously been reported.

Our results indicated significant proteins related to *Lacticaseibacillus rhamnosus*, such as relaxase and L-proline glycine betaine binding ABC transporter protein (ProX), which exhibited high VIP scores (Figure 4C). A high level of relaxase enzyme in fermented foods confers multiple benefits, including enhanced genetic adaptability through horizontal gene transfer; improved stress tolerance ensuring survival in acidic, salty, and oxidative environments; increased probiotic efficacy; improved gut adhesion and associated health benefits; and optimized fermentation efficiency, contributing to better food texture, flavor, and preservation [42,43]. Bacteria in fermented foods are often exposed to high osmotic stress due to salt, acid, and dehydration. To counter this, ProX facilitates the uptake of L-proline and glycine betaine, which serve as osmoprotectants, preventing dehydration and ensuring bacterial survival in high-salt environments such as cheese, kimchi, and fermented meats [44].

ShinyGO is a bioinformatic tool allowing researchers to perform gene ontology and pathway enrichment analyses, including fold enrichment calculations for proteins in various datasets such as those obtained from fermented foods. In this study, the ShinyGO analysis revealed the elevated expression of genes involved in DNA-related pathways (DNA repair, DNA damage response, and DNA replication) shared across all groups, indicating that these processes are fundamental responses to fermentation. Fermented foods are a source of bioactive compounds from probiotics and phytochemicals, which play a key role in enhancing DNA repair and preventing DNA damage in both probiotic strains and host cells [40]. These compounds include exopolysaccharides, short-chain fatty acids, polyphenols, flavonoids, and sulforaphane, all of which contribute to antioxidant defense, epigenetic regulation, and DNA stability [45]. The regular consumption of fermented vegetables may enhance both probiotic survival and genomic stability in the host. However, the protein biosynthesis pathway was uniquely observed in the vitexin group on day 15, suggesting that vitexin (flavone glycosides) might have a distinct effect on protein production by cells at this timepoint, possibly via its roles in maintaining cellular health, protecting against oxidative stress, and indirectly influencing protein synthesis [46].

A comparison of the six groups of fermented vegetables highlighted interesting trends in protein levels over time. On day 15, all three formulations were associated with significantly higher levels of proteins including aldo/keto reductase and aspartokinase than on day 0. Additionally, glycosyltransferases and the AAA family ATPase were found to be more abundant on day 0. These findings suggest potential areas for further research into how fermentation affects protein profiles over time. Aldo-keto reductases (AKRs) are a family of enzymes that play crucial roles in the metabolism of carbohydrates, lipids, and xenobiotics [47], contributing to the overall health benefits of probiotics found in fermented foods. These enzymes catalyze the reduction of aldehydes and ketones to their corresponding alcohols [47], which can influence the fermentation process and enhance the bioavailability of nutrients. A previous study highlighted the antioxidant properties of AKRs, showing that they can confer protective effects against oxidative stress in the gut microbiome [48]. The functional roles of AKRs in LGG-derived fermented foods underscore their importance in nutrition and health. Aspartokinase is an important enzymatic component involved in the biosynthetic pathway of essential amino acids, particularly those in the aspartate family, including lysine, threonine, methionine, and isoleucine, which is critical for growth and protein synthesis in LAB [49]. A previous study highlighted the metabolic pathways in *Lactobacillus* species, showing that aspartokinase plays a role in amino acid biosynthesis, which can impact the nutritional value of fermented foods [50]. Glycosyltransferases are enzymes that catalyze the transfer of sugar moieties to proteins or other molecules, playing a crucial role in protein glycosylation [51]. These enzymes facilitate the decomposition and metabolism of carbohydrates and proteins, facilitating the fermentation process and improving product flavor, particularly in plant-based fermented foods [52]. Another protein identified here is AAA family ATPase, which may play a role in stress responses and the maintenance of cellular homeostasis [53]. Research has suggested that fermentative bacteria such as LAB use AAA ATPases to regulate metabolic shifts, helping them survive and enhancing their fermentation efficiency [54]. As additional findings in this study, we also observed positive correlations of DNA-binding proteins with DPPH and FRAP ( Supplemetary Figure S5C). DNA-binding proteins regulate microbial proteolytic systems, which break down food proteins into bioactive peptides with antioxidant properties as well as certain microbes producing phenolic compounds, exopolysaccharides, and vitamins (e.g., B2, C, E), which enhance the antioxidant capacity [55].

In a further analysis of the findings related to *Levilactobacillus brevis*, we identified proteins with high VIP scores including Q03N47 (nickase), A0A7Z6MKU9 (protein-tyrosine phosphatase), and Q03U15 (alpha-glucosidase, family 31 of glycosyl hydrolases). Nickases are a class of endonucleases contributing to genome maintenance and stability, which also influence natural transformation and genetic exchange within microbial communities [56]. Nickase enzymes from LAB have attracted attention for their potential applications in food fermentation and biotechnology [57]. Meanwhile, protein-tyrosine phosphatases (PTPs) in LAB are involved in regulatory mechanisms such as nutrient sensing, transcriptional regulation, and stress responses [58]. Alpha-glucosidase is found in several LAB species, highlighting the diversity and potential of these bacteria in producing this enzyme [59]. The presence of α-glucosidase in LAB is crucial for the fermentation of various plant-based foods, contributing to the degradation of starch and the production of desirable flavors and textures [60]. Moreover, the results of the fold enrichment analysis indicated that arginine biosynthesis occurred in the three formulations of fermented vegetables on day 0. By day 15, the analysis revealed additional pathways, including antibiotic resistance in the probiotic group and repressor pathways in the vitexin group. These findings underscore the evolving nature of microbial interactions and metabolic pathways in fermented vegetables over time.

The analysis conducted through ANOVA indicated that the levels of nickase and protein-tyrosine phosphatase were significantly elevated in the three formulas at day 15 compared with the levels at day 0. This finding revealed important temporal changes in the concentrations of these proteins within the formulations. These elevations may indicate active bacterial metabolism and stress adaptation in response to the fermentation environment, potentially influencing the functional and nutritional properties of the formulas. We also found significant associations of uronate isomerase and helix-turn-helix domain-containing protein with TPC, DPPH, and ORAC. Given the functions of these proteins, these findings could reflect the polyphenol metabolism, stress responses, and the release of bioactive compounds, which could, in turn, improve the nutritional and functional properties of fermented products. In the case of uronate isomerase, it may contribute to overall antioxidant capacity by facilitating the breakdown of uronic acids, leading to the production of beneficial compounds that can scavenge free radicals and reduce oxidative stress. For example, polysaccharides that are abundant in uronic acid possess significant antioxidant properties. These properties can be attributed to the ability of uronic acid groups to donate hydrogen atoms, thereby playing a vital role in neutralizing free radicals [61]. Meanwhile, in the case of helix-turn-helix proteins like OxyR, these play a critical role in regulating the expression of antioxidant genes. By facilitating the binding of this protein to DNA, OxyR can modulate gene expression in response to oxidative stress, thereby contributing to cellular defense mechanisms [62]. However, thymidine kinase (TK) was found to be negatively correlated with TPC, DPPH, and ORAC. TK is crucial for DNA synthesis and cell proliferation, especially in rapidly growing microbes [63]. These conditions may result in the high production of metabolites such as ethanol and hydrogen peroxide by certain yeasts and LAB, which might indirectly cause oxidative stress [64].

This study presents a vital proteomic analysis of fermented foods, integrating multi-omics data and advanced tools for visualizing such data. By exploiting these capabilities in combination, this work contributes to a more comprehensive understanding of LAB metabolism, ultimately leading to improvements in the quality, functionality, and probiotic potential of fermented foods for health benefits. However, there are several limitations associated with this study, primarily related to methodological, biological, and analytical issues. The extraction and identification of LAB proteins pose significant challenges. Membrane-associated and low-abundance proteins are often difficult to extract and detect due to solubility issues and loss during sample preparation [65]. However, coupling fractionation techniques using LC-MS/MS can improve their sensitivity in detecting the proteins present at low levels [66]. Variability in LAB strains and fermentation conditions significantly influences protein expression. The same bacterial species may exhibit different proteomic profiles under various environmental conditions regarding factors such as pH, temperature, and substrate composition [43]. Standardizing fermentation parameters across different food formulas can minimize such variability. Furthermore, this work was affected by database and annotation limitations related to protein identification due to incomplete or poorly annotated genomes having been established for many LAB species, leading to identification errors. Additionally, peptide sequencing can help identify novel or uncharacterized proteins [67].

## 5. Conclusions

This proteomic analysis of selected genera and species of LAB in three formulas of fermented vegetables, assessed on the first day of production and 2 weeks later, revealed dynamic changes in protein expression over time, possibly reflecting the metabolic activity of the bacteria. An increase in specific functional proteins was observed, which has implications for the development of tailored fermentation processes, the optimization of starter cultures, and the creation of functional foods that promote gut health and overall wellbeing while also informing sustainable food production practices.

## Figures and Tables

**Table 1:** Five proteins from each group of fermented vegetables exhibited significant differences in their levels related to *Lactobacillus*, *Weissella*, *Lacticaseibacillus rhamnosus*, and *Levilactobacillus brevis*.

	Genus/Species	Protein Names	Tukey’s HSD (*p* Value < 0.05)
	*Lactobacillus*		
1	A0A0R2G7H2	Diadenosine tetraphosphatase-like protein	P15-N0; P15-N15; P15-P0; V0-P15; V15-P15
2	A0A7X9N5X7	GNAT family N-acetyltransferase	P15-N0; V15-N0; P15-N15; V15-N15; P15-P0; V15-P0; V0-P15; V15-P15; V15-V0
3	A0A1Z5IEV5	UDP-N-acetylglucosamine 1-carboxyvinyltransferase	N15-N0; P0-N0; P15-N0; V0-N0; V15-N0
4	A0A510WQG2	ATP-dependent Clp protease ATP-binding subunit ClpC	N15-N0; P15-N0; P0-N15; P15-N15; V0-N15; V15-N15; P15-P0; V0-P15; V15-P15
5	A0A0R2FNY3	Transposase	N15-N0; P15-N0; V0-N0; V15-N0; P0-N15; P15-P0; V0-P0; V15-P0
	***Weissella***		
1	A0A7X6LMH9	Aminoacyltransferase	N15-N0; P0-N0; P15-N0; V15-N0; P0-N15; V0-N15; P15-P0; V0-P0; V15-P0; V0-P15; V15-V0
2	A0A6C2CBJ3	ABC transporter permease	P15-N0; V15-N0; P15-N15; V15-N15; P15-P0; V15-P0; V0-P15; V15-V0
3	A0A2S1KT73	ATP-binding/permease protein CydD	P15-N0; P15-N15; P15-P0; V0-P15; V15-P15
4	A0A0D1M163	VWA-like domain-containing protein	N15-N0; P0-N0; P15-N0; V0-N0; V15-N0
5	A0A7L8CHR7	ABC transporter permease	P15-N0; V15-N0; P15-N15; V15-N15; P15-P0; V15-P0; V0-P15; V15-V0
	***Lacticaseibacillus rhamnosus***		
1	A0A249DF14	tRNA (guanine-N(1)-methyltransferase	P15-N0; V0-N0; P15-P0; V0-P0
2	A0A6N2XUY3	Capsid protein (F protein)	N15-N0; P15-N0; V15-N0; P0-N15; V0-N15; P15-P0; V15-P0; V0-P15; V15-V0
3	A0A6N2ZUA9	site-specific DNA-methyltransferase (adenine-specific)	N15-N0; P15-N0; V15-N0; P0-N15; P15-P0; V15-P0
4	A0A7Y7QFE1	Signal peptidase I	N15-N0; P0-N15; P15-N15; V0-N15; V15-N15
5	A0A7Y7QFS3	Aldo/keto reductase	N15-N0; P15-N0; V15-N0; P0-N15; V0-N15; P15-P0; V15-P0; V0-P15; V15-V0
	***Levilactobacillus brevis***		
1	A0A5B7Y3K0	RepC protein	N15-N0; P15-N0; V15-N0; P0-N15; V0-N15; P15-P0; V15-P0; V0-P15; V15-V0
2	Q03U02	Uronate isomerase	N15-N0; P15-N0; V15-N0; P0-N15; V0-N15; P15-P0; V15-P0; V0-P15; V15-V0
3	A0A5B7Y1U6	DNA helicase RecQ	P15-N0; V15-N0; P15-N15; V15-N15; P15-P0; V15-P0; V0-P15; V15-V0
4	Q03N47	Nickase	N15-N0; P15-N0; V0-N0; V15-N0; P0-N15; P15-P0; V0-P0; V15-P0
5	A0A7Z6MKU9	Protein-tyrosine-phosphatase	N15-N0; P15-N0; V15-N0; P0-N15; V0-N15; P15-P0; V15-P0; V0-P15; V15-V0

N0, P0, and V0; standard formula, probiotic formula, and vitexin-formula, respectively, at day 0; N15, P15, and V15—those groups at day 15.

## Data Availability

The original contributions presented in the study are included in the article/Supplementary Materials, further inquiries can be directed to the corresponding author.

## Supplementary Materials

The following supporting information can be downloaded at: https://www.mdpi.com/article/10.3390/foods14071148/s1.
