# Supplementary material for: Metaproteomic Analysis of Fermented Vegetable Formulations with Lactic Acid Bacteria: A Comparative Study from Initial Stage to 15 Days of Production
Source: Foods. 2025 Mar 26;14(7):1148. doi: 10.3390/foods14071148 (PMC11988878; doi:10.3390/foods14071148)
Supplement: Supplementary file 1 [file foods-14-01148-s001.zip › foods-3498453-Tables S1-S5 .pdf]

Supplementary Table S1. Relative abundance of taxonomies which demonstrated statistical significance after FDR adjustment among 6 groups of fermented vegetables; Standard formula on day 0 and day 15, probiotic formula on day 0 and day 15, and vitexin formula on day 0 and day 15.

**Fermented vegetable group**	**P-value**	**FDR**
Standard formula day 0 vs. day 15		
s__ Levilactobacillus brevis	1	1
s__ Lacticaseibacillus rhamnosus	1	1
g__Lactobacillus	1	1
g__Weissella	0.747	1
g__Pediococcus	0.023	0.243
Probiotic formula day 0 vs. day 15		
s__ Lacticaseibacillus rhamnosus	0.00276	0.0115
s__ Levilactobacillus brevis	1	1
g__Lactobacillus	0.212	0.737
g__Weissella	0.322	0.8
g__Pediococcus	0.000804	0.0153
Vitexin formula day 0 and day 15.		
s__ Levilactobacillus brevis	0.0000435	0.000155
s__ Lacticaseibacillus rhamnosus	1	1
g__Lactobacillus	0.000204	0.00645
g__Weissella	0.000871	0.0156
g__Pediococcus	0.148	0.563

Supplementary Table S2. Significant differences in protein expression relative to genus Lactobacillus among 6 groups of fermented vegetables (one-way ANOVA with post hoc test).

	Protein ID	Protein names	Tukey's HSD (p<0.05)	Box plot (comparison between 6 groups)
1	A0A0R2G7H2	Diadenosine tetraphosphatase-like protein	P15-N0; P15-N15; P15-P0; V0-P15; V15-P15	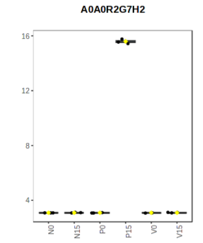
2	A0A7X9N5X7	GNAT family N-acetyltransferase	P15-N0; V15-N0; P15-N15; V15-N15; P15-P0; V15-P0; V0-P15; V15-P15; V15-V0	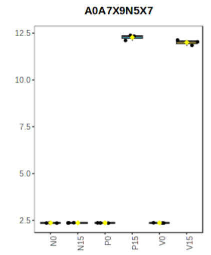
3	A0A1Z5IEV5	UDP-N-acetylglucosamine 1-carboxyvinyltransferase	N15-N0; P0-N0; P15-N0; V0-N0; V15-N0	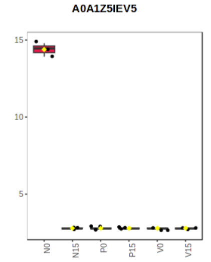
4	A0A510WQG2	ATP-dependent Clp protease	N15-N0; P15-N0; P0-N15; P15-N15; V0-N15; V15-N15; P15-P0; V0-P15; V15-P15	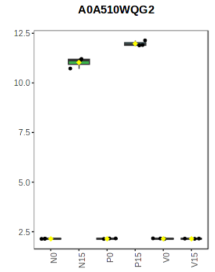
5	A0A0R2FNY3	Transposase	N15-N0; P15-N0; V0-N0; V15-N0; P0-N15; P15-P0; V0-P0; V15-P0	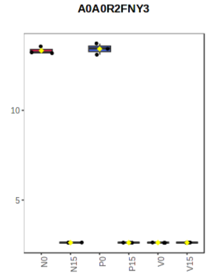
6	A0A386PY73	LysR family transcriptional regulator	P15-N0; P15-N15; P15-P0; V0-P15; V15-P15	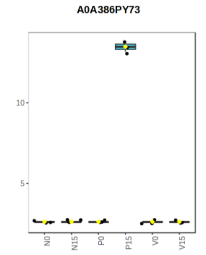

7	A0A3M6SEC8	Gram-positive cocci surface proteins LPxTG domain-containing protein	N15-N0; P15-N0; P0-N15; P15-N15; V0-N15; V15-N15; P15-P0; V0-P15; V15-P15	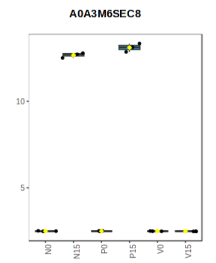
8	Q74I40	HTH cro/C1-type domain-containing protein	P15-N0; V15-N0; P15-N15; V15-N15; P15-P0; V15-P0; V0-P15; V15-V0	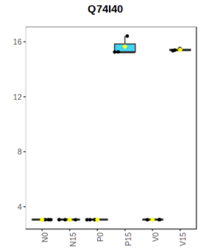
9	A0A2Z4W036	Ribonuclease Y (RNase Y)	V15-N0; V15-N15; V15-P0; V15-P15; V15-V0	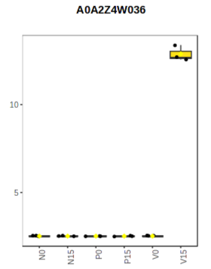
10	A0A0R2L0R0	Glyoxalase (Lactoylglutathione lyase)	P15-N0; V15-N0; P15-N15; V15-N15; P15-P0; V15-P0; V0-P15; V15-V0	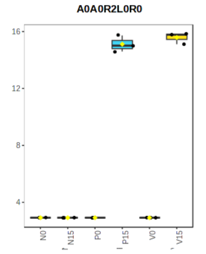
11	A0A5R8LUU0	ATP-binding cassette domain-containing protein	P0-N0; P0-N15; P15-P0; V0-P0; V15-P0	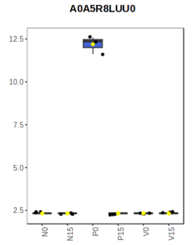
12	A0A4Z0JDU1	IS110 family transposase	N15-N0; P15-N0; V15-N0; P0-N15; V0-N15; P15-P0; V15-P0; V0-P15; V15-V0	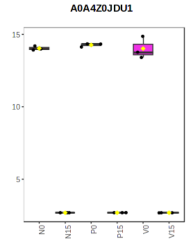
13	A0A0D6A210	DNA polymerase IV (Pol IV)	N15-N0; P0-N15; P15-N15; V0-N15; V15-N15	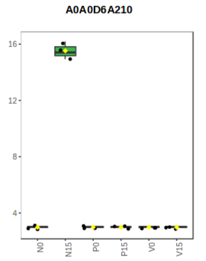

14	W6T759	Major facilitator transporter	P15-N0; P15-N15; P15-P0; V0-P15; V15-P15	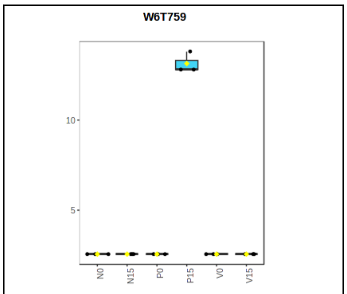
15	C7XVF8	cysteine desulfurase	N15-N0; P15-N0; V15-N0; P0-N15; V0-N15; V15-N15; P15-P0; V15-P0; V0-P15; V15-V0	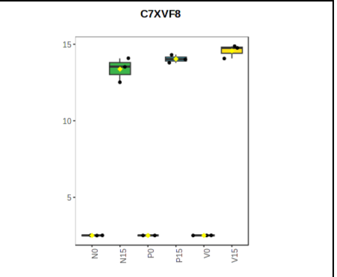
16	A0A244CCA7	Peptide O-xylosyltransferase	P15-N0; V15-N0; P15-N15; V15-N15; P15-P0; V15-P0; V0-P15; V15-P15; V15-V0	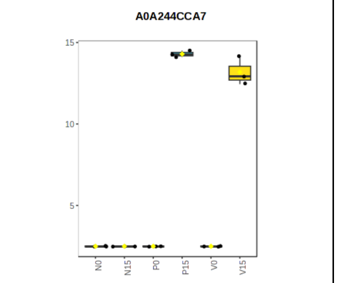
17	A0A0R2FL27	XRE family transcriptional regulator	N15-N0; P0-N0; V0-N0; V15-N0; P0-N15; P15-N15; P15-P0; V15-P0; V0-P15; V15-P15	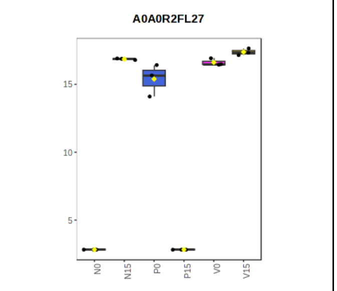
18	A0A2K9HG25	WxL domain-containing protein	P15-N0; V15-N0; P15-N15; V15-N15; P15-P0; V15-P0; V0-P15; V15-V0	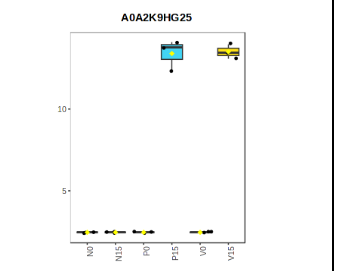
19	I7LCV4	Repressor of fructose operon	N15-N0; P15-N0; V15-N0; P0-N15; V0-N15; P15-P0; V15-P0; V0-P15; V15-V0	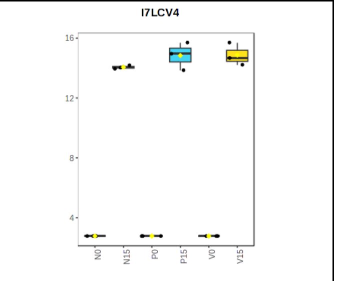
20	Q88YK2	Phosphatidylglycerol--prolipoprotein diacylglyceryl transferase	N15-N0; P0-N15; P15-N15; V0-N15; V15-N15	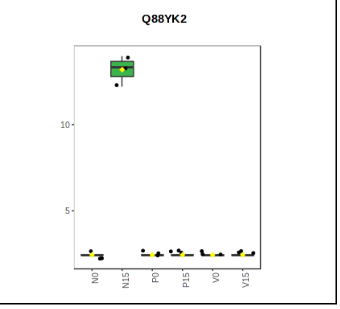

21	M5J4A7	D-alanine--D-alanine ligase	P0-N0; P0-N15; P15-P0; V0-P0; V15-P0	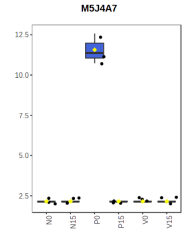
22	W6T4C8	2,5-diketo-D-gluconic acid reductase	N15-N0; P15-N0; V15-N0; P0-N15; V0-N15; P15-P0; V15-P0; V0-P15; V15-V0	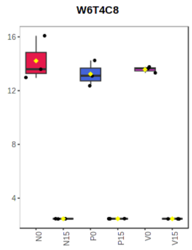
23	A0A5P1X6E6	Protein RecA (Recombinase A)	N15-N0; P0-N0; P15-N0; V0-N0; V15-N0	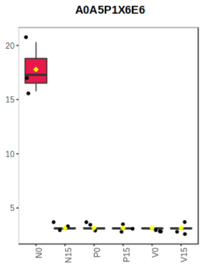
24	A0A9Q8QUS6	Polyphosphate kinase	N15-N0; P0-N0; P15-N0; V0-N0; V15-N0	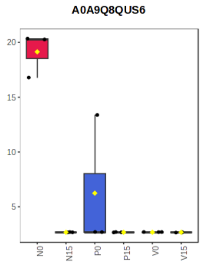
25	A0A4Q9Y834	MFS transporter	N15-N0; P15-N0; V15-N0; P0-N15; V0-N15; P15-P0; V15-P0; V0-P15; V15-V0	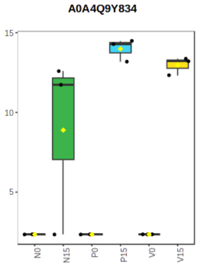
26	A0A6A8MB78	CopY/TcrY family copper transport repressor	N15-N0; P15-N0; P0-N15; V0-N15; V15-N15; P15-P0; V0-P15; V15-P15	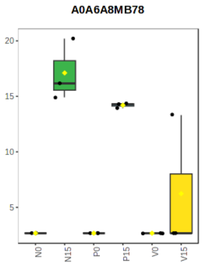
27	A0A2R3JUG2	Bifunctional oligoribonuclease/PAP phosphatase NrnA	N15-N0; P15-N0; V0-N0; V15-N0; P0-N15; P15-P0; V0-P0; V15-P0	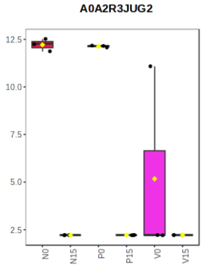

28	S4NSW7	Zn-dependent dehydrogenase	N15-N0; P15-N0; V15-N0; P0-N15; V0-N15; P15-P0; V15-P0; V0-P15; V15-V0	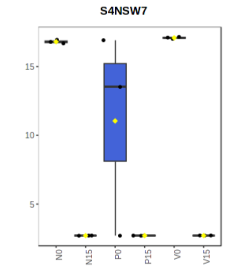
29	A0A7Z8G5Q8	XRE family transcriptional regulator	V0-N0; V0-N15; V0-P0; V0-P15; V15-V0	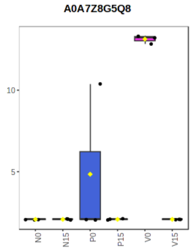
30	A0A162F141	Helix-turn-helix domain-containing protein	N15-N0; P15-N0; V15-N0; P0-N15; P15-P0; V15-P0	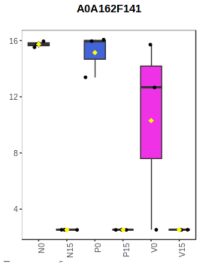
31	A0A0H4QJ62	DUF5658 domain-containing protein	N15-N0; P15-N0; V15-N0; P0-N15; V0-N15; P15-P0; V15-P0; V0-P15; V15-V0	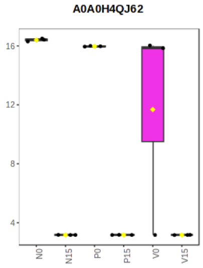
32	E1NRX3	Sortase family	N15-N0; P0-N0; P15-N0; V15-N0; V0-N15; V0-P0; V0-P15; V15-V0	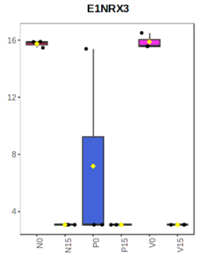
33	A0A0U5FAL1	Mobilization protein	N15-N0; P15-N0; V15-N0; P0-N15; P15-P0; V15-P0; V0-P15; V15-V0	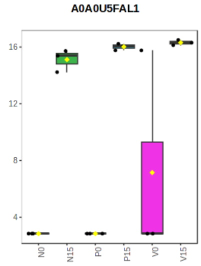

Supplementary Table S3. Significant differences in protein expression relative to genus Weissella among 6 groups of fermented vegetables (one-way ANOVA with post hoc test).

	Protein ID	Protein names	Tukey's HSD (p-value <0.05)	Box plot (comparison between 6 groups
1	A0A7X6LMH9	Aminoacyltransferase	N15-N0; P0-N0; P15-N0; V15-N0; P0-N15; V0-N15; P15-P0; V0-P0; V15-P0; V0-P15; V15-V0	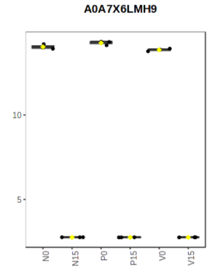
2	A0A6C2CBJ3	ABC transporter permease	P15-N0; V15-N0; P15-N15; V15-N15; P15-P0; V15-P0; V0-P15; V15-V0	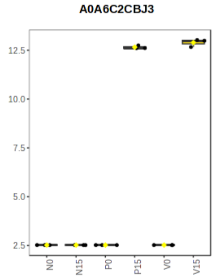
3	A0A2S1KT73	ATP-binding/permease protein CydD	P15-N0; P15-N15; P15-P0; V0-P15; V15-P15	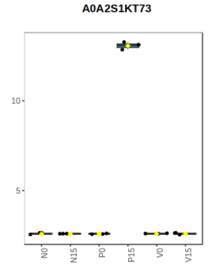
4	A0A0D1M163	VWA-like domain-containing protein	N15-N0; P0-N0; P15-N0; V0-N0; V15-N0	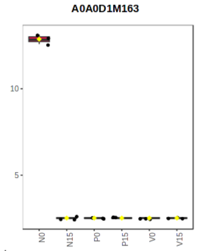
5	A0A7L8CHR7	ABC transporter permease	P15-N0; V15-N0; P15-N15; V15-N15; P15-P0; V15-P0; V0-P15; V15-V0	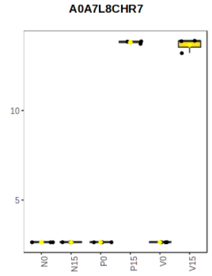
6	A0A9Q8JGD0	NgoFVII family restriction endonuclease	N15-N0; P15-N0; V15-N0; P0-N15; V0-N15; P15-P0; V15-P0; V0-P15; V15-V0	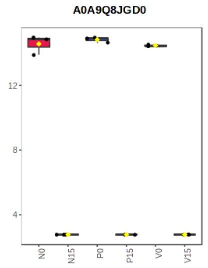

7	A0A380NVY9	Ribonuclease R	N15-N0; P15-N0; V0-N0; V15-N0; P0-N15; P15-P0; V0-P0; V15-P0	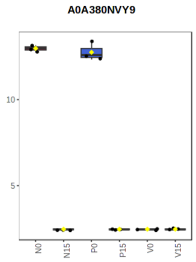
8	G0UEU2	Primosomal protein DnaI	N15-N0; P0-N0; V0-N0; V15-N0; P0-N15; P15-N15; P15-P0; V15-P0; V0-P15; V15-P15	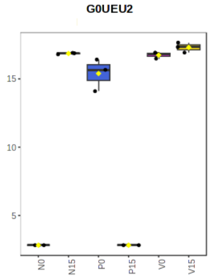
9	A0A7G9T6Z3	Sigma-70 family RNA polymerase sigma factor	N15-N0; P15-N0; V15-N0; P0-N15; V0-N15; P15-P0; V15-P0; V0-P15; V15-V0	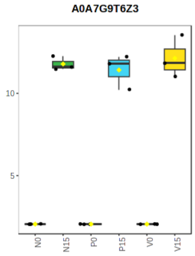
10	A0A0R2JJ73	Transcriptional regulator	N15-N0; P0-N15; P15-N15; V0-N15; V15-N15	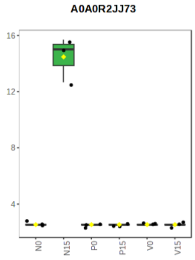
11	G0UGR8	Large ribosomal subunit protein uL11	N15-N0; P15-N0; V15-N0; P0-N15; P15-N15; V0-N15; P15-P0; V15-P0; V0-P15; V15-P15; V15-V0	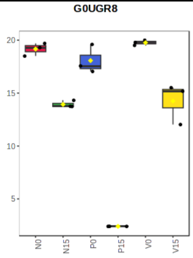
12	G0UIE7	DDE domain-containing protein	N15-N0; P15-N0; V15-N0; P0-N15; V0-N15; P15-P0; V15-P0; V0-P15; V15-V0	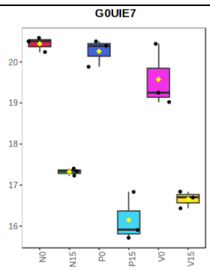
13	G0UF85	SseB protein N-terminal domain-containing protein	N15-N0; P15-N0; V15-N0; P0-N15; V0-N15; P15-P0; V15-P0; V0-P15; V15-V0	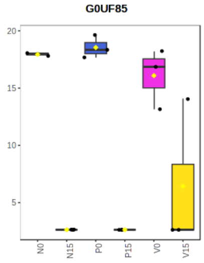

14	C5R9T8	Type I restriction modification DNA specificity domain protein	P15-N0; V15-N0; P15-N15; V15-N15; P15-P0; V15-P0; V0-P15; V15-V0	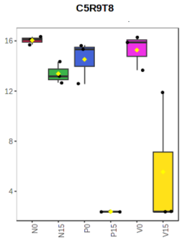
15	A0A7L8CH05	ABC transporter permease	P15-N0; V0-N0; V15-N0; P15-N15; V0-N15; V15-N15; P15-P0; V0-P0; V15-P0	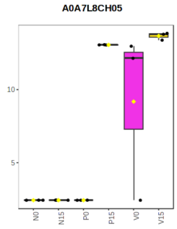
16	A0A923SN36	Tape measure protein	P15-N0; V15-N0; P15-P0; V15-P0; V0-P15; V15-V0	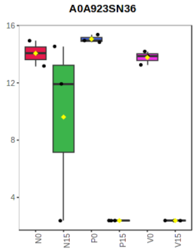
17	A0A9Q8N883	ABC transporter substrate-binding protein	P15-N0; V0-N0; P15-N15; V0-N15; P15-P0; V0-P0; V15-P15; V15-V0	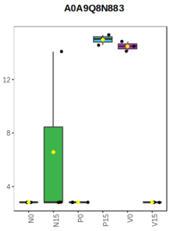
18	A0A6C2C525	Cardiolipin synthase (CL synthase)	N15-N0; P15-N0; P0-N15; V0-N15; P15-P0; V0-P15	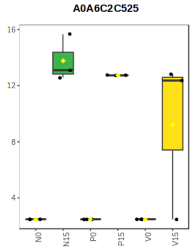
19	A0A6G8B144	RNA-binding transcriptional accessory protein	N15-N0; P15-N0; P0-N15; V0-N15; V15-N15; P15-P0; V0-P15; V15-P15	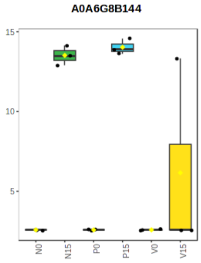
20	A0A6G8B0X9	Uridylate kinase (UK)	N15-N0; P15-N0; V0-N0; V15-N0; P0-N15; P15-P0; V0-P0; V15-P0	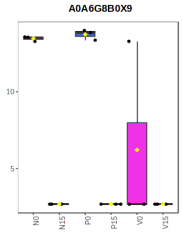
21	G0UF58	Aminopeptidase	N15-N0; P0-N0; P15-N0; V15-N0; V0-P15	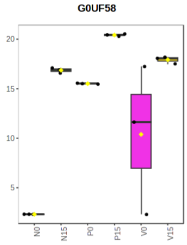

22	A0A7H1MMZ9	Aspartate--tRNA(Asp/Asn) ligase	N15-N0; P15-N0; V0-N0; V15-N0; P0-N15; P15-P0; V0-P0; V15-P0	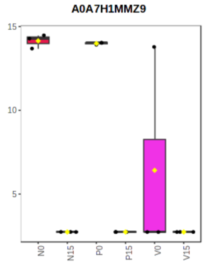
23	C5R8I8	Type I restriction modification DNA specificity domain protein	N15-N0; P15-N0; V15-N0; P0-N15; V0-N15; P15-P0; V15-P0; V0-P15; V15-V0	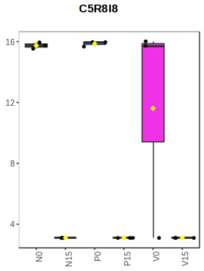
24	A0A923SP46	Glycosyl transferase family 28 C-terminal domain-containing protein	N15-N0; P15-N0; V0-N0; V15-N0; P0-N15; P15-P0; V0-P0; V15-P0	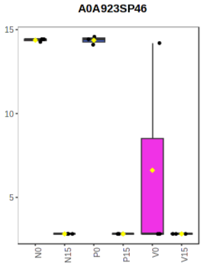
25	A0A9Q8JJE7	Putative hemin transport system permease protein HrtB	N15-N0; P15-N0; V15-N0; V0-N15; V15-P0; V0-P15; V15-V0	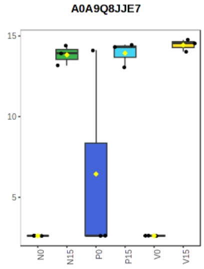
26	A0A6C2C4M0	SF3 helicase domain-containing protein	N15-N0; P15-N0; V0-N0; V15-N0; P0-N15; P15-P0; V0-P0; V15-P0	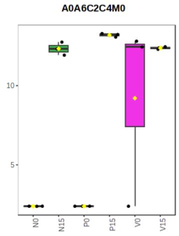
27	A0A9Q8NAP7	Cd(2+)-exporting ATPase	P15-N0; V15-N0; P15-N15; V15-N15; P15-P0; V15-P0; V0-P15; V15-V0	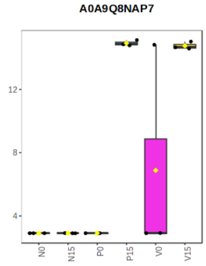
28	A0A9Q8JHG9	Glycosyltransferase	N15-N0; P15-N0; V15-N0; P0-N15; V0-N15; P15-P0; V15-P0; V0-P15; V15-V0	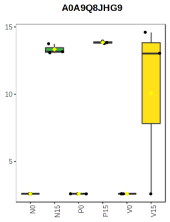

29	G0UG34	ABC superfamily ATP binding cassette transporter, membrane protein	N15-N0; P15-N0; V0-N0; V15-N0; P0-N15; P15-P0; V0-P0; V15-P0	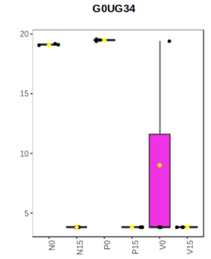
30	A0A9Q8N9Y7	DUF536 domain-containing protein	N15-N0; P15-N0; V15-N0; P0-N15; P15-P0; V15-P0; V0-P15	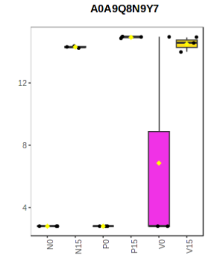

Supplementary Table S4. Significant differences in protein expression relative to *Lacticaseibacillus rhamnosus* among 6 groups of fermented vegetables (one-way ANOVA with post hoc test).

	Protein ID	Protein names	Tukey's HSD (p value <0.05)	Bo plot (comparison between 6 groups)
1	A0A249DF14	tRNA (guanine-N(1)-methyltransferase	P15-N0; V0-N0; P15-P0; V0-P0	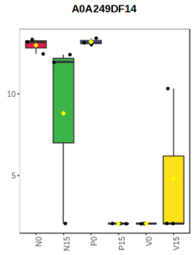
2	A0A6N2XUY3	Capsid protein (F protein)	N15-N0; P15-N0; V15-N0; P0-N15; V0-N15; P15-P0; V15-P0; V0-P15; V15-V0	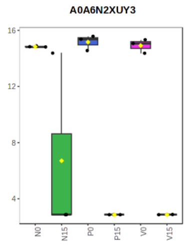
3	A0A6N2ZUA9	site-specific DNA-methyltransferase (adenine-specific)	N15-N0; P15-N0; V15-N0; P0-N15; P15-P0; V15-P0	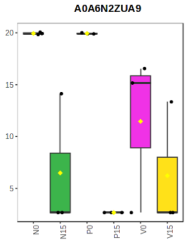
4	A0A7Y7QFE1	Signal peptidase I	N15-N0; P0-N15; P15-N15; V0-N15; V15-N15	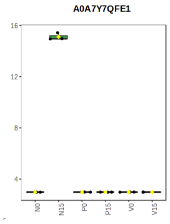
5	A0A7Y7QFS3	Aldo/keto reductase	N15-N0; P15-N0; V15-N0; P0-N15; V0-N15; P15-P0; V15-P0; V0-P15; V15-V0	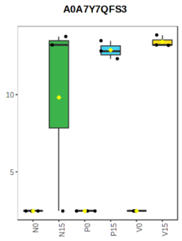
6	A0A7S7JI46	Glycosyltransferase	P15-N0; P0-N15; P15-P0; V15-P0	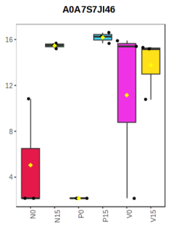
7	A0A7Y7QDC4	Aspartokinase	N15-N0; P15-N0; V15-N0; P0-N15; V0-N15; P15-P0; V15-P0; V0-P15; V15-V0	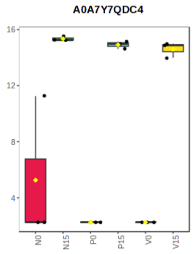

8	A0A7Y7QDK6	DNA-binding protein	P0-N0; P0-N15; P15-P0; V0-P0; V15-P0	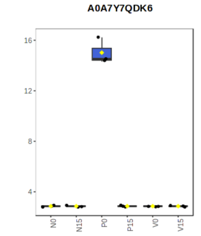
9	A0A7Y7QE37	GGDEF domain-containing protein	P0-N0; P0-N15; P15-P0; V0-P0	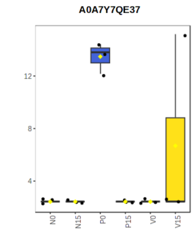
10	A0A7S7FNJ1	Phage minor tail protein	N15-N0; P15-N0; V0-N0; V15-N0; P0-N15; P15-P0; V0-P0; V15-P0	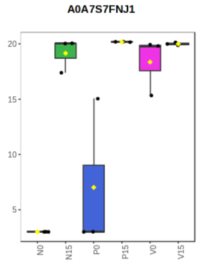
11	A0A7S7JGY4	Transposase	N15-N0; V15-N0; P15-N15; V15-P15	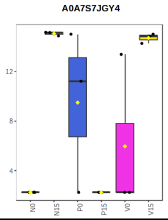
12	A0A7Y7QI88	PTS sugar transporter subunit IIA	V15-N0; V15-N15; V15-P0; V15-P15; V15-V0	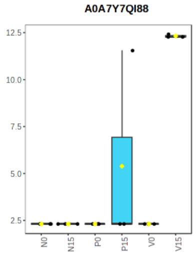
13	A0A7Y7UK12	PTS sugar transporter subunit IIB	P15-N0; V15-N0; P15-N15; V15-N15; P15-P0; V15-P0; V0-P15; V15-V0	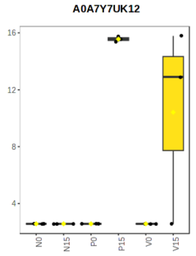
14	A0A7Y7UKC6	AAA family ATPase	P15-N0; P15-P0; V0-P15	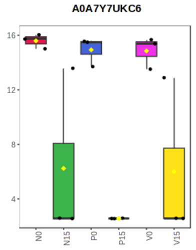
15	A0A7Y7UKL7	DNA-entry nuclease	P15-N0; V0-N0; V15-N0; P15-P0; V0-P0; V15-P0	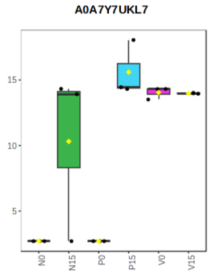

16	A0A809MTR5	Chloride channel protein	P15-N0; V15-N0; P15-N15; V15-N15; P15-P0; V15-P0; V0-P15; V15-V0	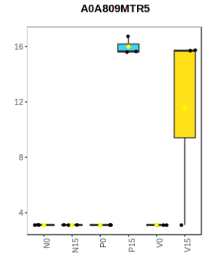
17	A0A809N1X8	Transcriptional regulator	P0-N0; P0-N15; P15-P0; V0-P0; V15-P0	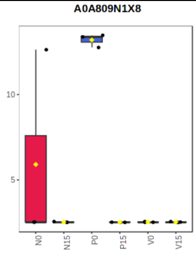
18	A0A830Q6L6	Transposase	N15-N0; P0-N0; P15-N0; V15-N0; V0-N15; V0-P0; V0-P15; V15-V0	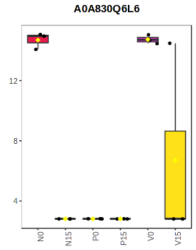
19	A0A853J5E0	Sugar fermentation stimulation protein homolog	N15-N0; P15-N0; V15-N0; P0-N15; P15-P0; V0-P0; V15-P0	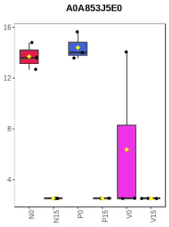
20	A0A853J5M3	IS66 family insertion sequence element accessory protein TnpB	P15-N0; V15-N0; V0-P15; V15-V0	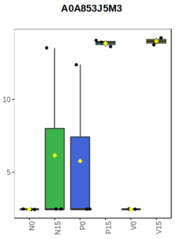
21	C7TE97	Glycosyltransferase	N15-N0; P15-N0; P0-N15; V0-N15; V15-N15; P15-P0; V0-P15; V15-P15	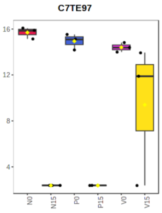
22	G8I2M7	Translation elongation factor Tu	P0-N0; V0-N0; P0-N15; V0-N15; P15-P0; V15-P0; V0-P15; V15-V0	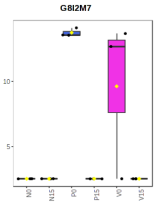
23	Q29ZJ2	PTS system lactose-specific EIICB component	P15-N0; P15-N15; P15-P0; V0-P15; V15-P15	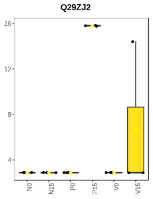

Supplementary Table S5. Significant differences in protein expression relative to *Levilactobacillus brevis* among 6 groups of fermented vegetables (One-way ANOVA with post hoc test)

	Protein ID	Protein names	Tukey's HSD ($p < 0.05$)	Box plot (comparison between 6 groups)
1	A0A5B7Y3K0	RepC protein	N15-N0; P15-N0; V15-N0; P0-N15; V0-N15; P15-P0; V15-P0; V0-P15; V15-V0	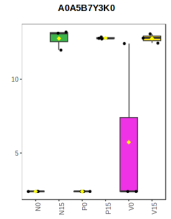
2	Q03U02	Uronate isomerase	N15-N0; P15-N0; V15-N0; P0-N15; V0-N15; P15-P0; V15-P0; V0-P15; V15-V0	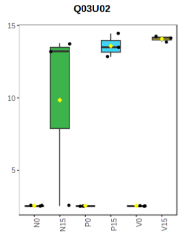
3	A0A5B7Y1U6	DNA helicase RecQ	P15-N0; V15-N0; P15-N15; V15-N15; P15-P0; V15-P0; V0-P15; V15-V0	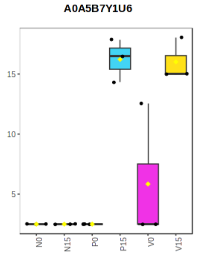
4	Q03N47	Nickase	N15-N0; P15-N0; V0-N0; V15-N0; P0-N15; P15-P0; V0-P0; V15-P0	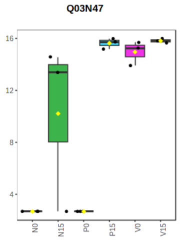
5	A0A7Z6MKU9	Protein-tyrosine-phosphatase	N15-N0; P15-N0; V15-N0; P0-N15; V0-N15; P15-P0; V15-P0; V0-P15; V15-V0	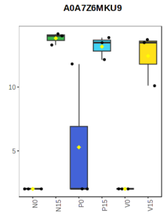
6	Q03SU1	Protein translocase subunit SecE	P15-N0; P15-N15; P15-P0; V0-P15; V15-P15	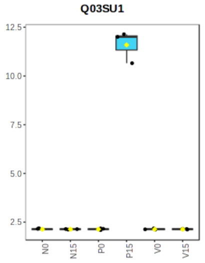
7	Q03N64	tRNA modification GTPase MnmE	N15-N0; P15-N0; V15-N0; P0-N15; V0-N15; P15-P0; V15-P0; V0-P15; V15-V0	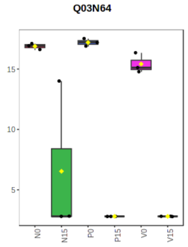

8	Q03NF7	Helix-turn-helix domain-containing protein	P15-N0; V15-N0; P15-N15; V15-N15; P15-P0; V15-P0; V0-P15; V15-V0	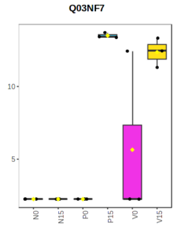
9	A0A2A3TUD8	LysM domain-containing protein	P0-N0; V0-N0; V15-N0; P0-N15; V0-N15; V15-N15; P15-P0; V0-P15; V15-P15	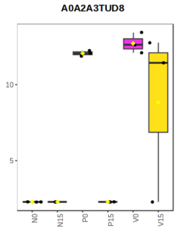
10	A0A7Z6MNT1	DUF4355 domain-containing protein	N15-N0; P15-N0; V15-N0; P0-N15; V0-N15; P15-P0; V15-P0; V0-P15	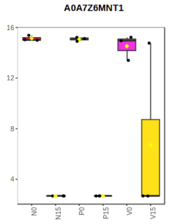
11	Q03QL7	Dihydrolipoyl dehydrogenase	N15-N0; P15-N0; V0-N0; P0-N15; V15-N15; P15-P0; V0-P0; V15-P15	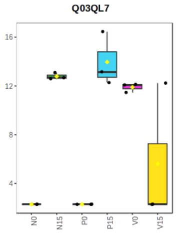
12	A0A7Z6MQW9	GIY-YIG nuclease family protein	N15-N0; P0-N0; V0-N0; P15-N15; P15-P0; V0-P15	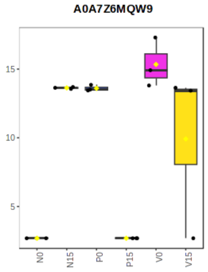
13	Q03U54	Glucose-6-phosphate 1-dehydrogenase (G6PD)	N15-N0; P15-N0; V0-N0; V15-N0; P0-N15; P15-P0; V0-P0; V15-P0	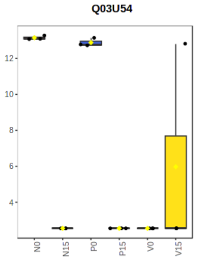
14	M5B0R2	Putative transposase InsK for insertion sequence element IS150	P15-N0; P15-N15; P15-P0; V0-P15; V15-P15	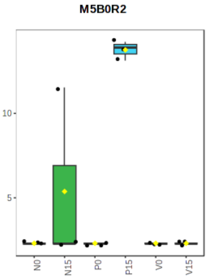
15	Q03QX6	Thymidine kinase	N15-N0; P15-N0; V0-N0; V15-N0; P0-N15; P15-P0; V0-P0; V15-P0	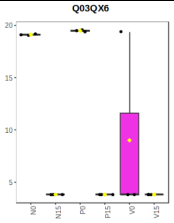

16	Q03U12	Galactose mutarotase related enzyme	P15-N0; P15-N15; P15-P0; V0-P15; V15-P15	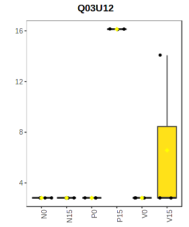
17	Q03RS3	Methionyl-tRNA formyltransferase	N15-N0; P15-N0; V15-N0; P0-N15; P15-P0; V15-P0	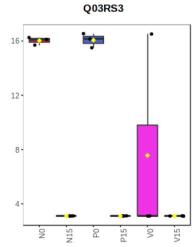
18	J7GT55	Penicillin-binding protein	N15-N0; P15-N0; V0-N0; V15-N0; P0-N15; P15-P0; V0-P0; V15-P0	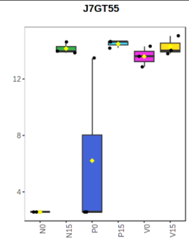
19	A0A5B7XXN0	Beta-galactosidase (Beta-gal)	N15-N0; P15-N0; V15-N0; P0-N15; V0-N15; P15-P0; V15-P0; V0-P15; V15-V0	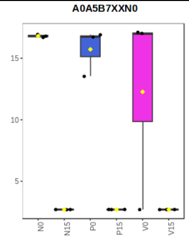
20	A0A0D0GQB8	Phosphoenolpyruvate-protein phosphotransferase	N15-N0; P15-N0; P0-N15; V0-N15; P15-P0; V0-P15; V15-V0	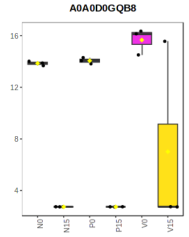
