# Supplementary figures and images for: Metaproteomic Analysis of Fermented Vegetable Formulations with Lactic Acid Bacteria: A Comparative Study from Initial Stage to 15 Days of Production
Source: Foods. 2025 Mar 26;14(7):1148. doi: 10.3390/foods14071148 (PMC11988878; doi:10.3390/foods14071148)

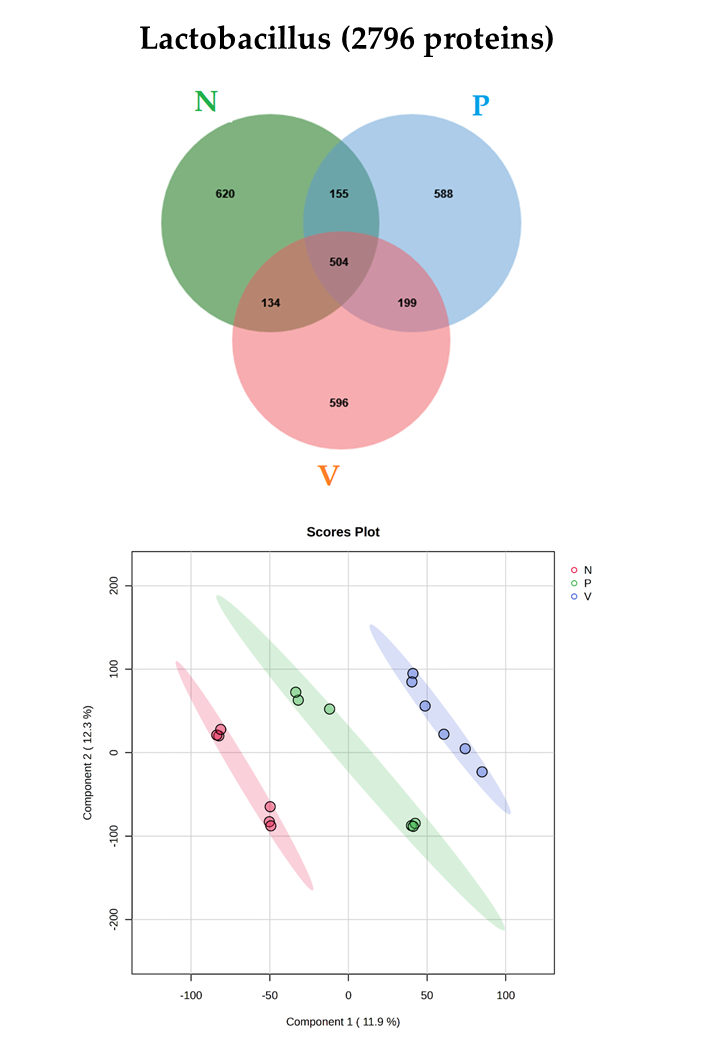

Supplement: Supplementary file 1 [file foods-14-01148-s001.zip › Supplementary Figure S1.TIF]

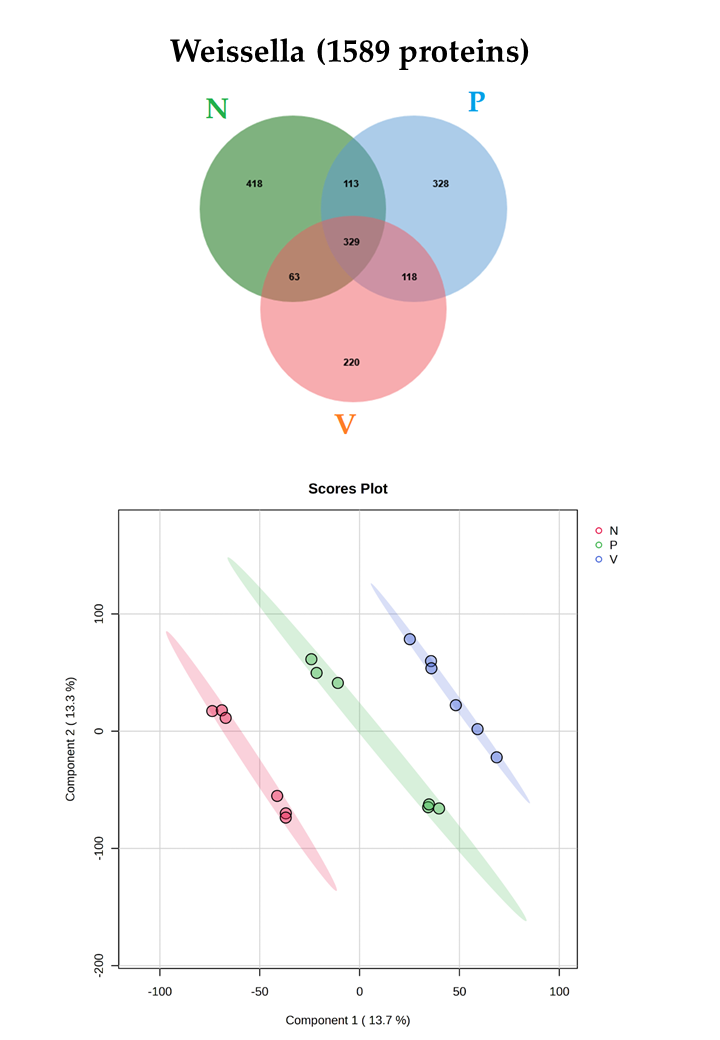

Supplement: Supplementary file 1 [file foods-14-01148-s001.zip › Supplementary Figure S2.TIF]

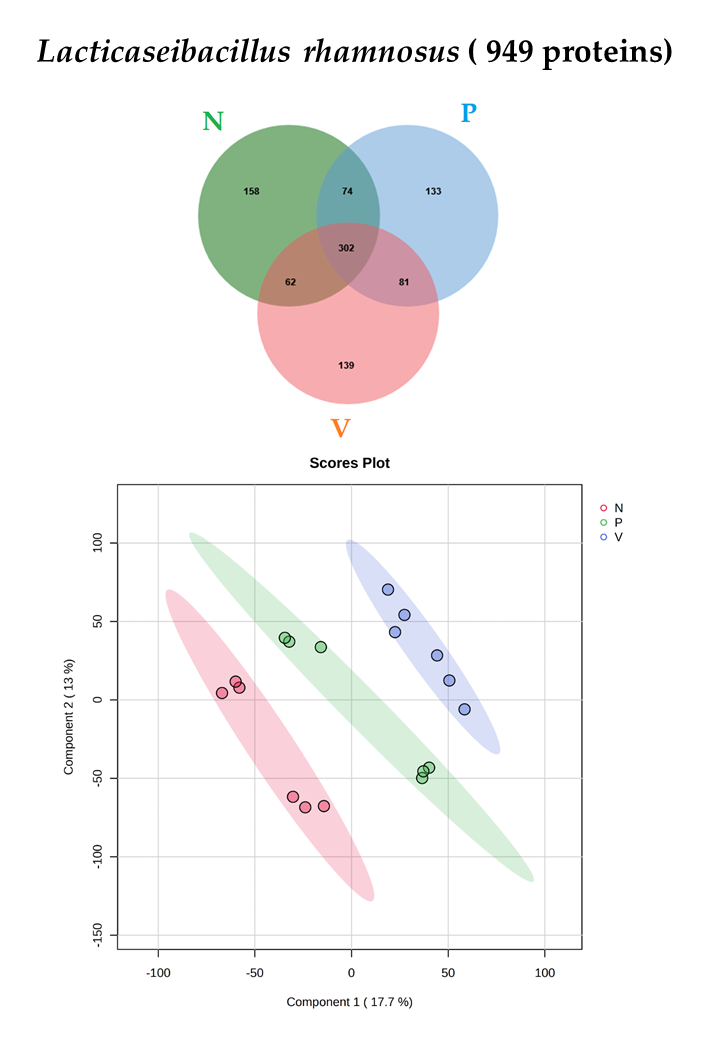

Supplement: Supplementary file 1 [file foods-14-01148-s001.zip › Supplementary Figure S3.TIF]

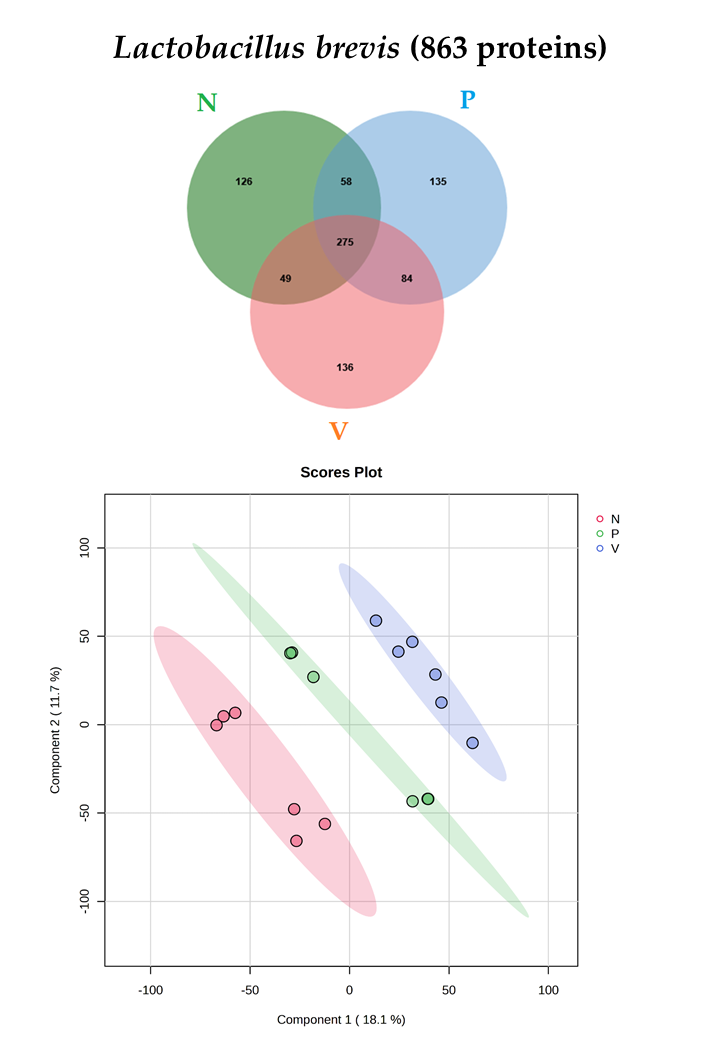

Supplement: Supplementary file 1 [file foods-14-01148-s001.zip › Supplementary Figure S4.TIF]

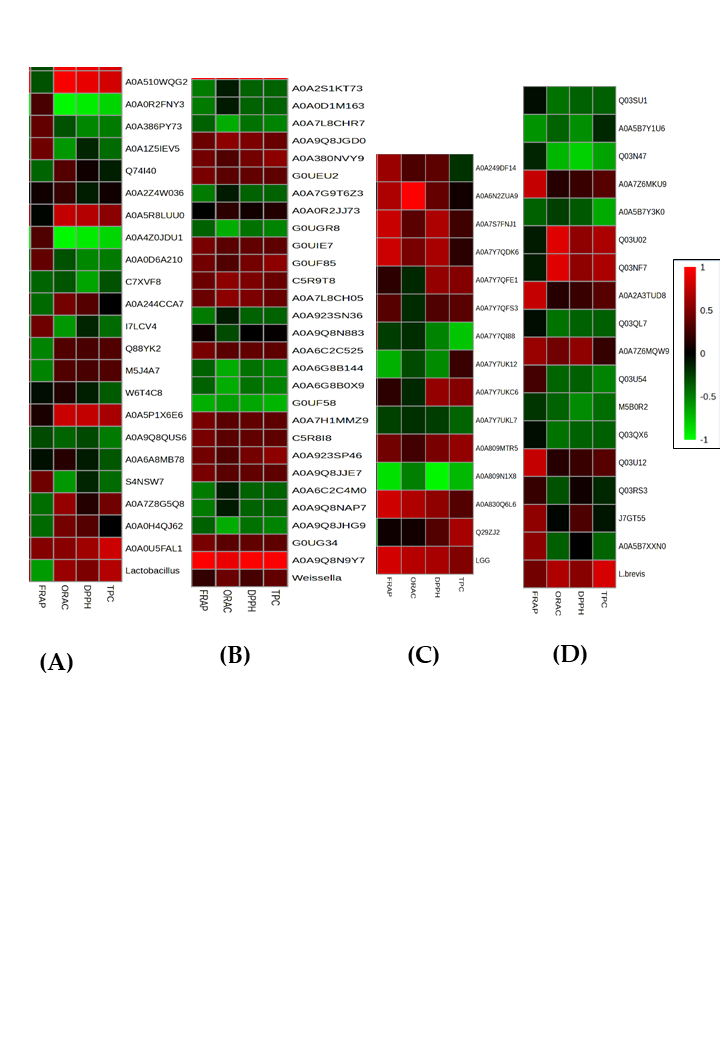

Supplement: Supplementary file 1 [file foods-14-01148-s001.zip › Supplementary Figure S5.tif]
